# Supplementary material for: Iontronic click-to-release enables electrically controlled delivery of drugs and biomolecules beyond charge and size limitations
Source: Nat Commun. 2026 Mar 31;17:4629. doi: 10.1038/s41467-026-70985-0 (PMC13199424; doi:10.1038/s41467-026-70985-0)
Supplement: Supplementary file 1 — Supplementary Information [file 41467_2026_70985_MOESM1_ESM.pdf]

SUPPORTING INFORMATION

Iontronic Click-to-Release Enables Electrically Controlled Delivery of Drugs and Biomolecules Beyond Charge and Size Limitations

Sebastian Hecko[+,1], Marle E.J. Vleugels[+,2], Christian Bayer[+,3], Donghak Byun[2], Moa E. Hörberg[2], Nikolaus Poremba[1], Rassen Boukraa[2], Patrick Keppel[1], Andreas Löffler[1], Walter Kuba[1], Helena Saarela Unemo[2], Iwona Bernacka Wojcik[2], Theresia Arbring Sjöström[2], Magnus Berggren[2,4], Daniel T. Simon[2], Rainer Schindl[3], Linda Waldherr[*,3,5], Hannes Mikula[*,1], Johannes Bintinger[*,1,2]

Affiliation:

[1] Institute of Applied Synthetic Chemistry, TU Wien, 1060 Vienna, Austria

[2] Laboratory of Organic Electronics, Department of Science and Technology, Linköping University, 601 74 Norrköping, Sweden

[3] Gottfried Schatz Research Center – Medical Physics and Biophysics, Medical University of Graz, 8010 Graz, Austria

[4] Wallenberg Initiative Materials Science for Sustainability, Department of Science and Technology, Linköping University, 601 74 Norrköping, Sweden

[5] BioTechMed-Graz, Austria, 8036 Graz, Austria

[+] These authors contributed equally

* Corresponding authors: linda.waldherr@medunigraz.at, hannes.mikula@tuwien.ac.at, johannes.bintinger@tuwien.ac.at

Supporting Information Contents

1 General methods ... 2

2 Synthesis ... 3

3 Click-to-release kinetics ... 7

3.1 Click kinetics ... 7

3.2 Release kinetics ... 8

4 Iontronic pump assembly ... 10

5 Iontronic delivery ... 12

5.1 Instruments and materials ... 12

5.2 Iontronic delivery in constant-bias mode ... 12

5.3 Iontronic delivery in step-function mode ... 14

6 Iontronic release of CA4 from 4 on cells ... 15

6.1 CA4 release after iontronic Tz delivery ... 15

6.2 CA4 efficacy ... 16

6.3 Potential-time traces ... 17

7 Iontronic release of CA4 from 6 on beads ... 18

8 Iontronic release of BSA from beads ... 20

8.1 Potential-time traces ... 20

8.2 SDS-PAGE ... 21

8.3 Background BSA Release Observed During K^+ Delivery ... 22

9 Cyclic voltammetry (CV) ... 23

9.1 Instruments and sample preparation ... 23

9.2 CV measurement ... 23

10 NMR Spectra ... 24

11 References ... 30

1 General methods

Unless otherwise noted, reactions were carried out under an atmosphere of argon in air-dried glassware with magnetic stirring. Air- and/or moisture-sensitive liquids were transferred via syringe. All reagents were purchased from commercial sources without further purification. **2Pyr$_2$** (3,6-bis(2-pyridyl)tetrazine) and **4MU** (4-methylumbelliferone) were obtained from Sigma-Aldrich, **CA4** (Combretastatin A-4) was obtained from BLD Pharmatech. **BSA**-N_3 was obtained from Vector Laboratories. Phosphate Buffered Saline (PBS, pH 7.4) was obtained from Sigma-Aldrich. THF and CH_2Cl_2 were dried using PURESOLV-columns (Inert Corporation). Dry DMF, dry DMSO were obtained from ACROS Organics and Sigma-Aldrich, respectively. Solvents used for reactions and column chromatography were purchased from Donau Chemie AG.

Column chromatography was performed using a BUCHI Sepacore Flash System (2 x BUCHI Pump Module C-605, BUCHI Pump Manager C-615, BUCHI UV Photometer C-635, and BUCHI Fraction Collector C-660) and a Pure Chromatography Flash/Prep Purification System C-850 (BUCHI). Silica gel 60 (40-63 µm) was obtained from Merck. Reversed phase preparative HPLC was done using a Phenomenex Kinetex AXIA LC column (C18 or C8; 5 µm, 100 Å) or a Phenomenex Luna column (C18; 10 µm, 100 Å). HPLC-grade solvents were purchased from Donau Chemie AG.

^{1}H and ^{13}C NMR spectra were recorded on a Bruker Ascend 600 MHz spectrometer at 20 °C. Chemical shifts are reported in ppm (δ) relative to tetramethylsilane and calibrated using solvent residual peaks. Data are shown as follows: Chemical shift, multiplicity (s = singlet, d = doublet, t = triplet, q = quartet, quint = quintet, m = multiplet, b = broad signal), coupling constants (*J*, Hz) and integration.

HPLC-MS (LCMS) analysis was performed on a Nexera X2 system (Shimadzu) comprised of LC-30AD pumps, a SIL-30AC autosampler, a CTO-20AC column oven, and a DGU-20A$_{5/3}$ degasser module. Detection was done using an SPD-M20A photodiode array and an LCMS-2020 mass spectrometer (ESI/APCI). If not stated otherwise, all separations were performed using a Waters XSelect CSH™ C18 2.5 µm (3.0 x 50 mm) column XP at 40 °C and a flow rate of 1.7 mL/min with 0.1% aqueous formic acid or ammonium formate buffer (2.5 mM, pH 8.4) and acetonitrile (gradient elution). Acidic HPLC conditions (acetonitrile/0.1% formic acid) 0 min: 5%, 0.15 min: 5%, 2.20 min: 98%, 2.50 min: 98%; Buffered HPLC conditions (acetonitrile/2.5 mM ammonium formate buffer, pH 8.4) 0 min: 5%, 0.15 min: 5%, 2.20 min: 98%, 2.50 min: 98%.

HRMS analysis of aqueous or non-aqueous (acetonitrile, methanol) solutions of the compounds (concentration: 10 ppm) was carried out on an Agilent 6230 LC TOFMS or Agilent 6545 Q-TOF mass spectrometer, both equipped with an Agilent Dual AJS ESI-Source. Data evaluation was performed using Agilent MassHunter Workstation Qualitative Analysis 10.0. Identification was based on peaks obtained from extracted ion chromatograms (extraction width ± 20 ppm).

2 Synthesis

Aminoethyl Tz (**1**)[1], rTCO-*p*-nitrophenol (rTCO-*p*NP)[2], sulfo-cTCO-DMEDA-CA4 (**4**)[3], cTCO-bis-NHS (**5**)[4] and CA4-*p*NP[3] were prepared according to known procedures.

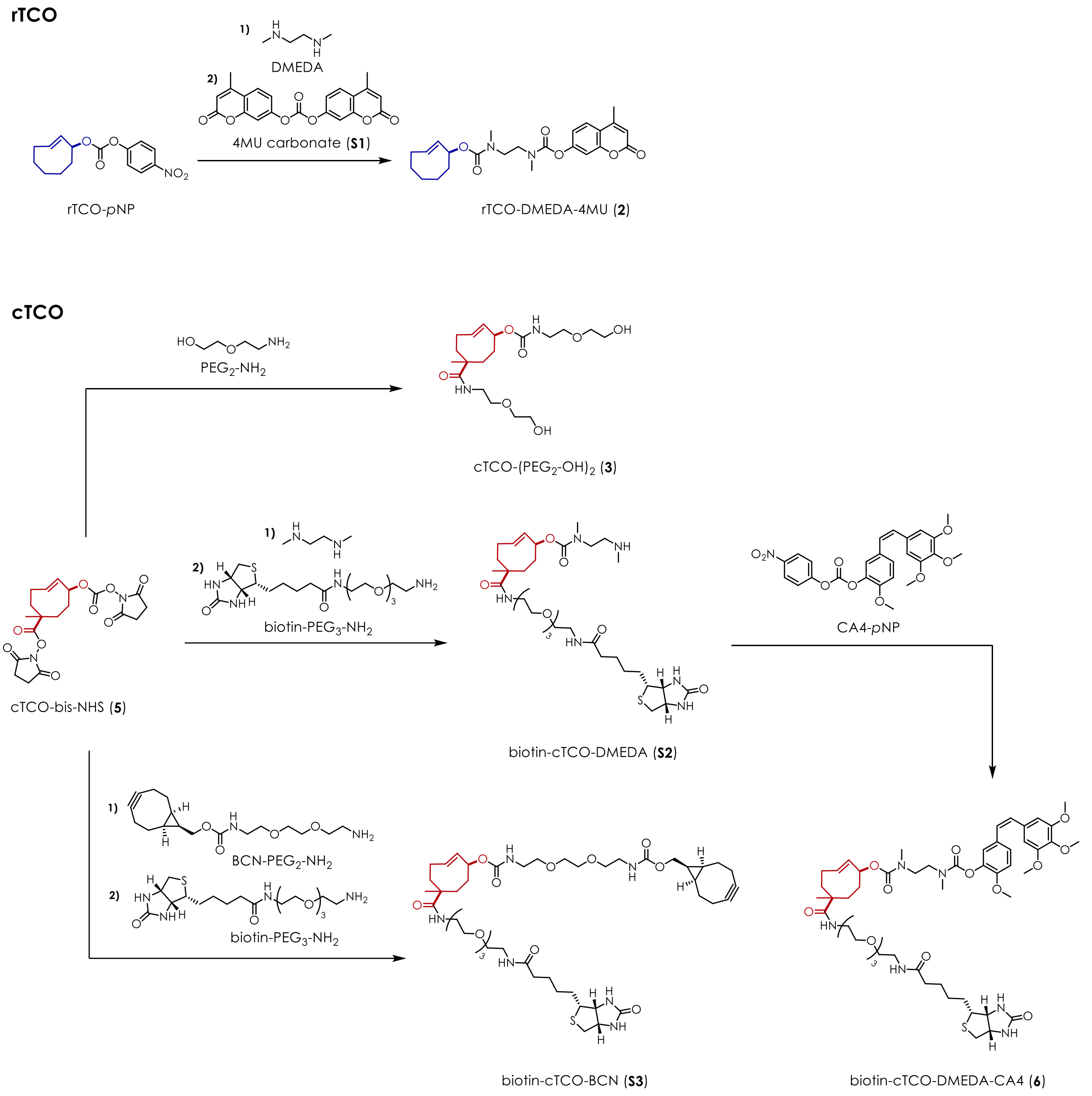

4MU carbonate (S1)

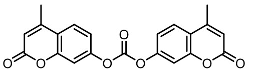

A three-necked round bottom flask was loaded with **4MU** (600 mg, 3.0 mmol, 2.0 eq.) dissolved in 25 mL dry THF, and cooled with an ice bath for 10 min. Triethylamine (516 mg, 5.1 mmol, 3.4 eq.) dissolved in 3 mL THF was added under argon, and the mixture was stirred for 5 min, followed by the addition of triphosgene (297 mg, 1.0 mmol, 0.7 eq.). The reaction was stirred for 16 h at room temperature. Subsequently, the reaction mixture was diluted with CH_2Cl_2 and washed three times with NH_4Cl solution, water, and brine each. The aqueous layer was re-extracted with CH_2Cl_2. A white precipitate formed and was filtered off. The combined organic layers were dried over Na_2SO_4, and the solvent was evaporated. The crude product was flashed with CH_2Cl_2/MeOH (10%) over silica to yield the desired product (**S1**) as a colorless solid (380 mg, 67%).

^{1}H NMR (600 MHz, CD_2Cl_2) δ 7.71 (s, 1H), 7.70 (s, 1H), 7.32 (d, J = 2.3 Hz, 2H), 7.28 (dd, J = 8.7, 2.4 Hz, 2H), 6.28 (q, J = 1.3 Hz, 2H), 2.45 (d, J = 1.3 Hz, 6H).

^{13}C NMR (151 MHz, CD_2Cl_2) δ 159.9, 154.2, 152.7, 151.9, 150.8, 125.9, 118.6, 117.0, 114.9, 109.7, 53.8, 53.6, 53.4, 53.3, 53.1, 18.5.

HRMS $[M+H]^+$ calcd. 379.0812 for $C_{21}H_{15}O_7^+$, found 379.0807 – Δ = 1.32 ppm.

rTCO-DMEDA-4MU (2)

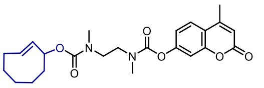

rTCO-*p*-nitrophenol (33 mg, 113 µmol, 1.0 eq., axial isomer) was dissolved in 5 mL CH_2Cl_2 and added *via* a syringe pump (30 µL/min) to a solution of *N*,*N*'-dimethyl-1,2-ethanediamine (DMEDA, 100 µL, 920 µmol, 8.0 eq.) in 2.5 mL DMF/CH_2Cl_2 (v/v 1:4) at 0 °C over 2 h. The mixture was stirred for 60 min at room temperature before the solvent was removed under vacuum to give the crude rTCO-DMEDA intermediate which was used without further purification. The residue was dissolved in CH_2Cl_2 and 4MU-carbonate (**S1**, 64 mg, 150 µmol, 1.5 eq.) was added, followed by addition of DIPEA (45 µL, 250 µmol, 2.5 eq.) and DMF (0.5 mL). The suspension turned immediately fluorogenic under UV irradiation and was stirred for 16 h. The solvent was subsequently removed under vacuum, and the crude product was purified by reversed phase column chromatography (C18, MeCN/H_2O gradient elution) to yield the desired product (**2**) as colorless solid (26 mg, 52%).

^{1}H NMR (600 MHz, CD_2Cl_2) δ 7.59 (ddd, *J* = 11.8, 8.5, 2.8 Hz, 1H), 7.13 – 7.05 (m, 2H), 6.19 (s, 1H), 5.86 – 5.70 (m, 1H), 5.53 (t, *J* = 15.5 Hz, 1H), 3.69 – 3.56 (m, 2H), 3.55 – 3.41 (m, 3H), 3.10 (d, *J* = 10.7 Hz, 2H), 3.01 (dd, *J* = 12.7, 6.1 Hz, 4H), 2.94 (d, *J* = 7.6 Hz, 2H), 2.11 – 1.88 (m, 4H), 1.83 (tt, *J* = 14.4, 7.2 Hz, 1H), 1.73 – 1.54 (m, 3H), 1.51 – 1.42 (m, 1H), 1.13 – 0.99 (m, 1H), 0.85 – 0.73 (m, 1H).

^{13}C NMR (151 MHz, CD_2Cl_2) δ 160.73, 160.70, 156.0, 155.9, 155.59, 155.57, 154.56, 154.53, 154.49, 154.4, 154.32, 154.29, 154.1, 154.0, 153.9, 152.57, 152.56, 132.1, 132.0, 132.0, 131.9, 125.68, 125.65, 125.62, 125.56, 118.7 118.52, 118.45, 118.4, 117.8, 117.7, 117.6, 114.5, 114.42, 114.40, 114.37, 110.7, 110.5, 110.4, 75.0, 74.9, 74.8, 74.7, 48.0, 47.8, 47.5, 47.4, 47.3, 47.2, 46.7, 46.5, 41.2, 41.1, 41.0, 36.39, 36.36, 36.21, 36.18, 36.16, 35.8, 35.64, 35.62, 35.5, 35.4, 35.2, 35.0, 34.6, 29.5, 29.4, 24.8, 24.7, 24.5, 18.9.

*Compound **2** is reported as a mixture of rotamers.*

HRMS $[M+H]^+$ calcd. 443.2177 for $C_{24}H_{31}N_2O_6^+$, found 443.2203 – Δ = 5.86 ppm.

cTCO-(PEG$_2$-OH)$_2$ (3)

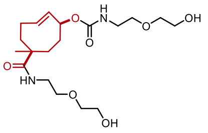

cTCO-bis-NHS (**5**) (10.2 mg, 24 µmol, 1.0 eq., axial isomer) was dissolved in dry DMSO (0.5 mL) and PEG$_2$-NH$_2$ (10 mg, 96 µmol, 4.0 eq.) was added in one portion. The reaction mixture was stirred at room temperature for 15 min and subsequently diluted with water (2 mL). Purification by reversed phase column chromatography (C18, H_2O/MeCN gradient elution, 0.1% formic acid) gave the desired product (**3**) as colorless oil (8.8 mg, 92%).

^{1}H NMR (600 MHz, CD_2Cl_2) δ 6.01 (s, 1H), 5.90 (ddd, *J* = 15.6, 11.1, 3.6 Hz, 1H), 5.60 (dd, *J* = 16.6, 2.6 Hz, 1H), 5.46 (d, *J* = 6.7 Hz, 1H), 5.12 (s, 1H), 3.72 – 3.67 (m, 4H), 3.58 – 3.50 (m, 8H), 3.40 – 3.35 (m, 4H), 2.52 (s, 1H), 2.25 (dtt, *J* = 27.4, 11.8, 5.8 Hz, 2H), 2.08 (td, *J* = 13.0, 4.7 Hz, 1H), 2.01 (s, 1H), 1.89 (dd, *J* = 15.6, 12.2 Hz, 1H), 1.84 – 1.74 (m, 2H), 1.70 (s, 1H), 1.55 (dd, *J* = 15.5, 6.5 Hz, 1H), 1.08 (s, 3H).

^{13}C NMR (151 MHz, CD_2Cl_2) δ 180.8, 156.1, 131.9, 131.8, 72.7, 72.6, 72.6, 70.4, 70.3, 62.0, 46.1, 44.7, 41.2, 39.7, 36.2, 31.6, 31.4, 18.2.

HRMS $[M+Na]^+$ calcd. 425.2258 for $C_{19}H_{34}N_2O_7Na^+$, found 425.2268 – Δ = 2.36 ppm.

biotin-cTCO-DMEDA (S2)

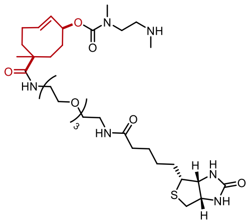

N,*N'*-Dimethyl-1,2-ethanediamine (9.8 mg, 114 µmol, 10.0 eq.) was dissolved in dry DMSO (114 µL, 1 M) and a solution of cTCO-bis-NHS (**5**) (4.8 mg, 11.4 µmol, 1.0 eq., axial isomer) in dry DMSO (114 µL, 0.1 M) was added dropwise in 2 min. The reaction mixture was stirred at room temperature for 30 min, after which biotin-PEG$_3$-NH$_2$ (9.6 mg, 22.8 µmol, 2.0 eq.) in dry DMSO (228 µL, 0.1 M) was added. The solution was stirred for 1 h and then diluted with a 1:1 mixture of 0.1% aqueous formic acid and MeCN (0.5 mL). The solution was directly loaded onto the C18 column and purified via reversed phase column chromatography (C18, H_2O/MeCN gradient elution, 0.1% formic acid) to obtain the desired product (**S2**) as colorless oil (2.5 mg, 31%).

^{1}H NMR (600 MHz, methanol-d$_4$) δ 8.54 (s, 2H), 5.92 (s, 1H), 5.71 (dd, *J* = 16.7, 2.6 Hz, 1H), 5.20 (s, 1H), 4.49 (ddd, *J* = 7.9, 5.1, 1.0 Hz, 1H), 4.31 (dd, *J* = 7.9, 4.5 Hz, 1H), 3.67 – 3.58 (m, 12H), 3.53 (dt, *J* = 11.5, 5.6 Hz, 5H), 3.38 – 3.32 (m, 4H), 3.24 – 3.16 (m, 3H), 3.09 (s, 3H), 2.97 (s, 1H), 2.93 (dd, *J* = 12.7, 5.0 Hz, 1H), 2.76 – 2.67 (m, 3H), 2.29 (td, *J* = 9.3, 3.8 Hz, 2H), 2.22 (t, *J* = 7.4 Hz, 2H), 2.20 – 2.13 (m, 1H), 2.06 – 1.97 (m, 2H), 1.96 – 1.89 (m, 1H), 1.81 – 1.53 (m, 6H), 1.50 – 1.39 (m, 2H), 1.14 (s, 3H).

^{13}C NMR (151 MHz, methanol-d$_4$) δ 183.4, 176.1, 166.1, 158.1, 132.9, 132.3, 75.0, 71.6, 71.3, 71.2, 70.6, 70.5, 63.4, 61.6, 57.0, 46.9, 46.6, 45.6, 41.1, 40.5, 40.3, 36.9, 36.7, 35.0, 34.0, 32.4, 31.9, 29.8, 29.5, 18.4.

HPLC-MS $[M+H]^+$ calcd. 699.41 for $C_{33}H_{59}N_6O_8S^+$, found 699.35.

biotin-cTCO-DMEDA-CA4 (6)

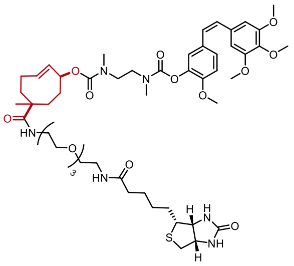

biotin-cTCO-DMEDA (**S2**) (2.6 mg, 3.7 µmol, 1.0 eq.) was dissolved in dry DMF (100 µL) and CA4-*p*NP (3.6 mg, 7.4 µmol, 2.0 eq.) was added followed by HOBt (1.0 mg, 7.4 µmol, 2.0 eq.) and DIPEA (11 µL, 114 µmol, 3.0 eq.). The reaction mixture was stirred overnight at room temperature and subsequently diluted with water (100 µL). Purification by reversed phase column chromatography (C18, H_2O/MeCN gradient elution, 0.1% formic acid) gave the desired product (**6**) as reddish oil (1.5 mg, 39%).

^{1}H NMR (600 MHz, methanol-d$_4$) δ 8.55 (s, 0.4H), 7.17 – 7.09 (m, 1H), 7.07 – 6.94 (m, 2H), 6.61 – 6.55 (m, 2H), 6.49 (s, 2H), 5.84 (dt, *J* = 28.3, 12.6 Hz, 1H), 5.64 (t, *J* = 17.9 Hz, 1H), 5.11 (s, 0.36H), 5.06 (s, 0.18H), 5.00 (s, 0.26H), 4.48 (dd, *J* = 7.8, 5.0 Hz, 1H), 4.29 (dd, *J* = 7.9, 4.5 Hz, 1H), 3.89 (s, 0.61H), 3.85 (s, 0.25H), 3.81 (s, 3H), 3.76 – 3.73 (m, 3H), 3.68 (d, *J* = 2.6 Hz, 6H), 3.65 – 3.55 (m, 12H), 3.55 – 3.49 (m, 5H), 3.47 (t, *J* = 5.7 Hz, 1H), 3.42 (m, 1H), 3.35 (m, 2H), 3.19 (ddd, *J* = 8.7, 5.9, 4.5 Hz, 1H), 3.13 (s, 0.36H), 3.09 (s, 0.36H), 3.05 (s, 0.27H), 3.03 (d, *J* = 6.0 Hz, 2H), 2.98 (s, 1H), 2.96 (s, 1H), 2.93 (s, 1H), 2.91 (dd, *J* = 12.7, 5.1 Hz, 1H), 2.70 (d, *J* = 12.7 Hz, 1H), 2.21 (t, *J* = 7.5 Hz, 2H), 2.27 – 2.12 (m, 2H), 2.12 – 1.80 (m, 4H), 1.77 – 1.54 (m, 6H), 1.43 (p, *J* = 7.8 Hz, 2H), 1.36 – 1.26 (m, 1H), 1.14 – 1.05 (m, 3H).

HRMS $[M+Na]^+$ calcd. 1063.5032 for $C_{52}H_{76}N_6O_{14}SNa^+$, found 1063.5063 – Δ = 2.91 ppm.

biotin-cTCO-BCN (S3)

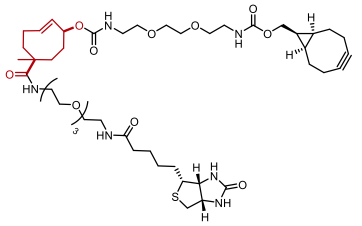

BCN-PEG$_2$-NH$_2$ (4 mg, 12.5 µmol, 1.0 eq.) in dry DMSO (125 µL, 0.1 M) was added to cTCO-bis-NHS (**5**) (5.3 mg, 12.5 µmol, 1.0 eq., axial isomer) dissolved in dry DMSO (125 µL, 0.1 M). The reaction mixture was stirred at room temperature for 30 min, after which biotin-PEG$_3$-NH$_2$ (5.8 mg, 13.8 µmol, 1.1 eq.) in dry DMSO (138 µL, 0.1 M) was added. The solution was stirred for 30 min and then diluted with a 1:1 mixture of water and MeCN (2 mL). The solution was directly loaded onto the C18 column and purified via reversed phase column chromatography (C18, H_2O/MeCN gradient elution) to obtain the desired product (**S3**) as colorless solid (2.5 mg, 31%).

^{1}H NMR (600 MHz, CD_2Cl_2) δ 6.38 (s, 1H), 6.10 (s, 1H), 5.88 – 5.76 (m, 2H), 5.58 (s, 1H), 5.53 (d, *J* = 16.6 Hz, 1H), 5.41 (s, 1H), 5.04 (s, 1H), 5.00 (s, 1H), 4.41 (t, *J* = 5.8 Hz, 1H), 4.22 (qd, *J* = 4.5, 2.0 Hz, 1H), 4.06 (d, *J* = 8.1 Hz, 2H), 3.55 – 3.50 (m, 14H), 3.49 – 3.42 (m, 8H), 3.32 (p, *J* = 5.3 Hz, 2H), 3.27 (p, *J* = 5.2 Hz, 6H), 3.08 (td, *J* = 7.4, 4.5 Hz, 1H), 2.84 (dd, *J* = 12.8, 5.0 Hz, 1H), 2.63 (d, *J* = 12.8 Hz, 1H), 2.25 – 2.07 (m, 12H), 2.05 – 1.96 (m, 1H), 1.89 (d, *J* = 14.4 Hz, 1H), 1.83 – 1.79 (m, 1H), 1.77 – 1.61 (m, 6H), 1.61 – 1.42 (m, 7H), 1.36 (p, *J* = 7.5 Hz, 3H), 1.31 – 1.23 (m, 1H), 1.00 (s, 3H), 0.85 (t, *J* = 9.9 Hz, 3H).

^{13}C NMR (151 MHz, CD_2Cl_2) δ 180.7, 173.2, 163.7, 157.1, 156.1, 132.0, 131.8, 99.1, 72.5, 70.8, 70.8, 70.6, 70.5, 70.40, 70.38, 70.2, 70.1, 63.0, 62.1, 60.5, 55.9, 54.2, 54.0, 53.8, 53.7, 53.5, 46.1, 44.6, 41.3, 41.2, 41.0, 39.7, 39.6, 36.30, 36.29, 36.1, 31.6, 31.4, 29.5, 28.5, 28.4, 26.0, 21.7, 20.5, 18.2, 18.1.

HRMS $[M+Na]^+$ calcd. 957.4978 for $C_{46}H_{74}N_6O_{12}SNa^+$, found 957.4984 – Δ = 0.69 ppm.

3 Click-to-release kinetics

3.1 Click kinetics

For second-order rate constant determination using stopped-flow analysis, Tz **1** was dissolved in DMSO at a concentration of 10 mM. A stock solution of cTCO-$(PEG_2\text{-}OH)_2$ (**3**) was prepared in DMSO at a concentration of 100 mM. The exact concentration of the TCO stock solution was determined by absorbance titration with 3,6-bis(2-pyridyl)tetrazine (**2Pyr₂**, Sigma-Aldrich) in DMSO using a Thermo Fisher Scientific NanoDrop One^C Microvolume UV-Vis Spectrophotometer in cuvette mode at 25 °C.

The TCO stock solution was diluted with DMSO to reach a concentration of 1 mM and then spiked with an excess of **$2Pyr_2$** stock solution (20.4 mM). Upon IEDDA reaction, the remaining tetrazine absorbance at 535 nm was measured. This procedure was repeated twice (standard addition) to determine the exact TCO stock concentration.

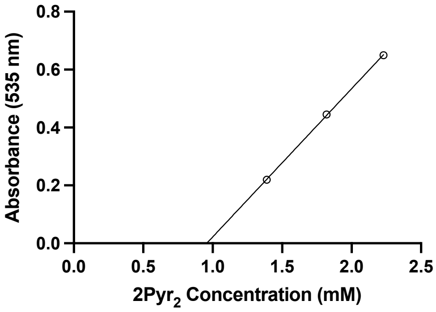

Based on the determined exact concentration, the initial DMSO stock solution of cTCO-$(PEG_2\text{-}OH)_2$ (**3**) was diluted into PBS (pH 7.4) or FluoroBrite DMEM + 10% FBS to reach a final concentration of 500 µM (<1% DMSO), whereas the tetrazine stock solution was diluted to a concentration of 100 µM.

Stopped-flow measurements were performed using an SX20-LED stopped-flow spectrophotometer (Applied Photophysics) equipped with a 535 nm LED (optical path length 10 mm, full width at half-maximum 34 nm) to monitor the characteristic tetrazine absorbance at 520-540 nm. The reagent syringes were loaded with solutions of **3** (500 µM) and **1** (100 µM) and the instrument was primed. Measurements were performed at 37 °C in sextuplicate and recorded automatically at the time of acquisition. Concentrations of the used solutions are mixed 1:1 in the analysis.

Data sets were analyzed by exponential fitting (one-phase association) using Prism 10 (GraphPad) to calculate the observed pseudo-first-order rate constants that were converted into the second-order rate constants by dividing by the concentration of the excess TCO compound. The second-order rate constant (k_2) of cTCO-$(PEG_2\text{-}OH)_2$ (**3**) with Tz **1** was determined to be **46 ± 3 M^{-1} s^{-1}** in PBS (pH 7.4) and **37 ± 2 M^{-1} s^{-1}** in FluoroBrite DMEM + 10% FBS, respectively. Uncertainties of TCO titration and stopped-flow analysis were evaluated experimentally from the dispersion of repeated measurements. The reported uncertainty is an expanded uncertainty calculated using a coverage factor of 2, resulting in a level of confidence of approximately 95%.[5]

Kinetic data with exponential fits
37 °C, DMSO content: <1%

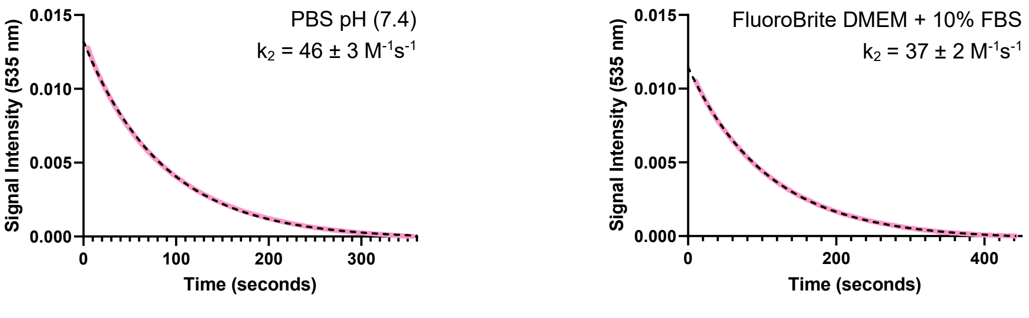

Fig. S1 Kinetic data (pink curves, n = 6 independent experiments) with exponential fits (dashed lines) of Tz **1** reacting with cTCO-$(PEG_2\text{-}OH)_2$ (**3**) in aqueous media at 37 °C.

3.2 Release kinetics

Reaction monitoring of the release experiment was performed on a Nexera X2® UHPLC system (Shimadzu®) with a temperature-controlled autosampler at 37 °C. For acidic HPLC conditions, the aqueous solvent was prepared by addition of 2.5 mL of neat formic acid to 2.5 L of HPLC-grade water to yield a final concentration of 0.1% formic acid. A stock solution of sulfo-cTCO-DMEDA-CA4 (**4**) was prepared at a concentration of 10 mM in DMSO. The exact concentration of the TCO stock solution was determined by absorbance titration (535 nm) as described in 3.1. A stock solution of Tz **1** was prepared at a concentration of 10 mM in DMSO.

For release kinetics measurements the stock solution of sulfo-cTCO-DMEDA-CA4 (**4**, 5.24 µL) was added to PBS (985 µL, containing 8.6% DMSO), and the click-to-release reaction was initiated by addition of the stock solution of Tz **1** (10 µL) to obtain starting concentrations of 50 µM TCO **4** and 100 µM Tz **1** (in 10% DMSO/PBS). The samples were immediately incubated at 37 °C in the autosampler and subjected to serial HPLC analysis in intervals of 30 min. The measurements were conducted in triplicate.

PDA data was collected for all samples. Relative quantification of intermediates and products was done using extracted chromatograms (wavelength: 254 nm). In addition, released **CA4** was quantified via external calibration. A **CA4** stock solution (20 mM) was prepared in DMSO and diluted with PBS (containing 10% DMSO) to reach a concentration of 100 µM. **CA4** standard solutions (1 µM - 75 µM) were prepared by serial dilution into PBS (containing 10% DMSO). All measurements were conducted in triplicate.

Selected chromatograms and MS data

Tz **1** triggered cleavage of sulfo-cTCO-DMEDA-CA4 (**4**)

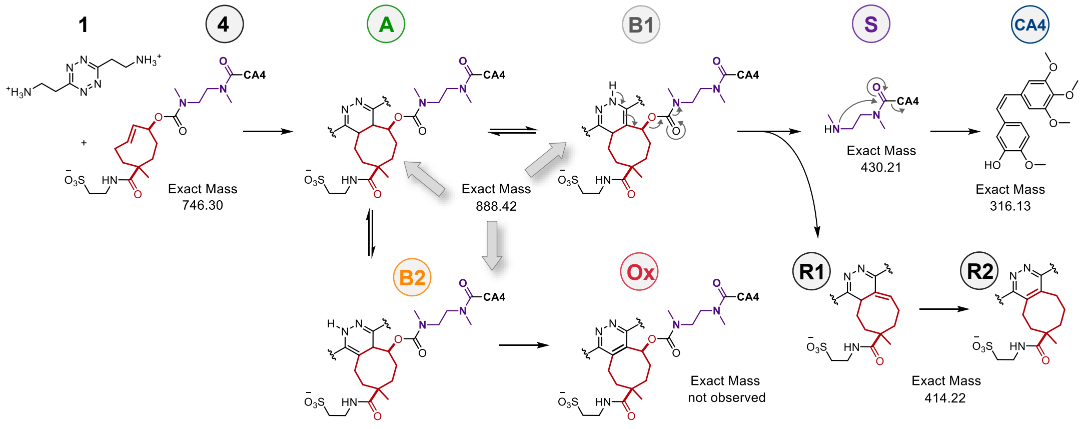

sulfo-cTCO-DMEDA-CA4 (**4**) + Tz **1**, 35 min reaction time, 58% **CA4** release

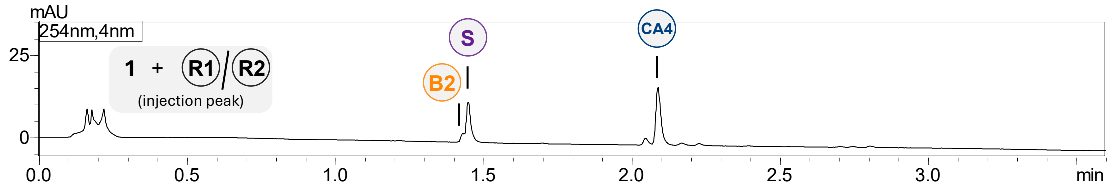

125 min reaction time, 92% **CA4** release

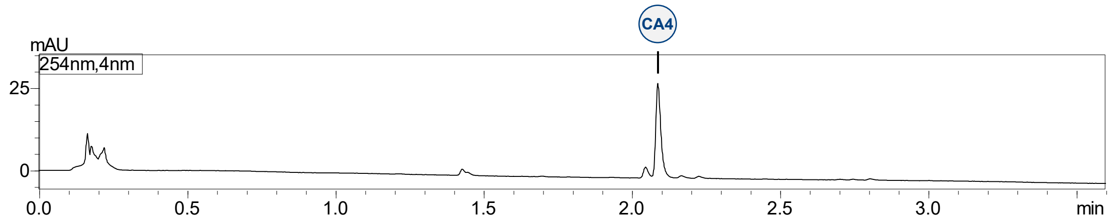

HPLC gradient (% acetonitrile in 0.1% formic acid) 0 min: 5%, 0.15 min: 5%, 3.20 min: 98%, 3.50 min: 98%.

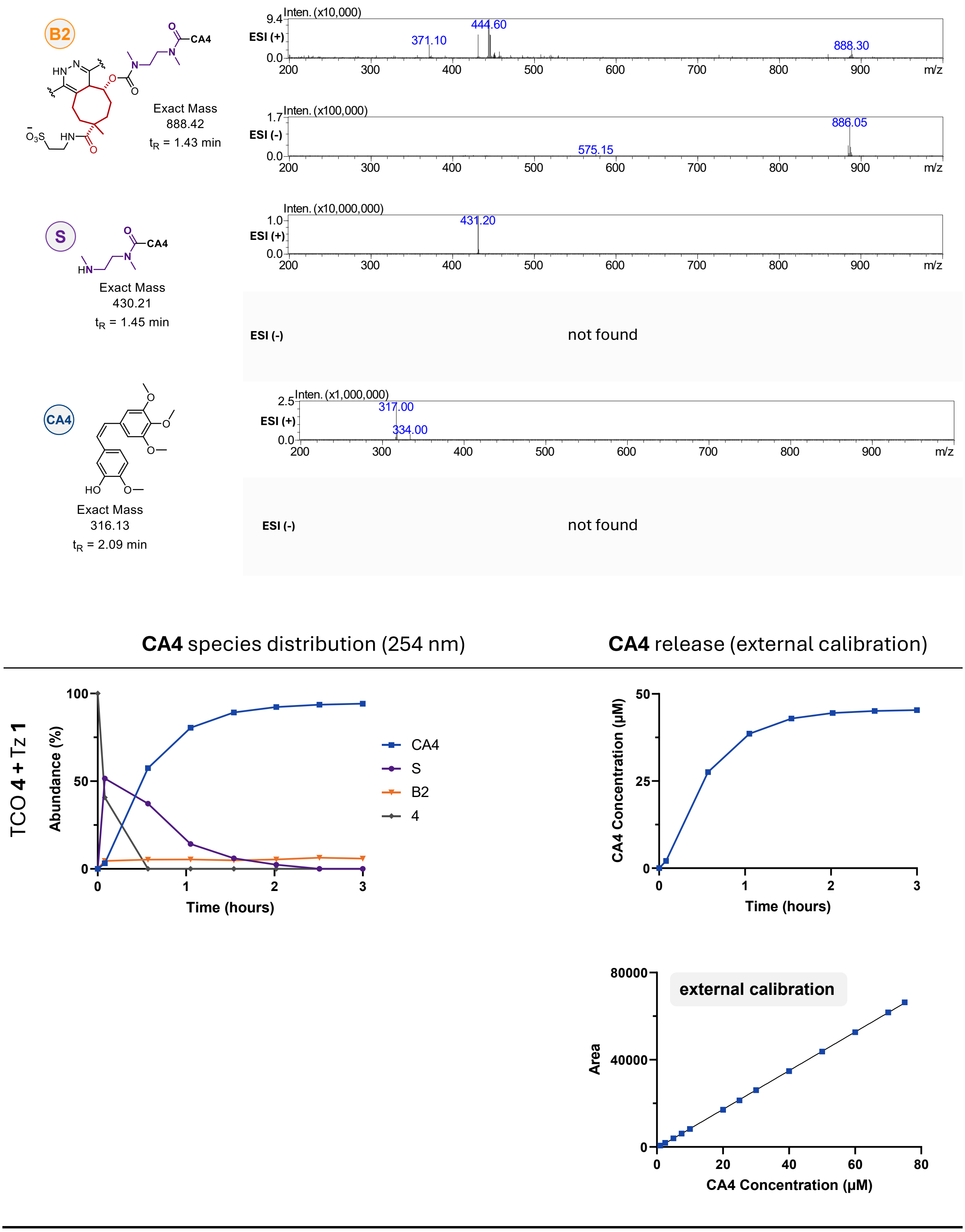

Fig. S2 Release performance of sulfo-cTCO-DMEDA-CA4 (**4**) (50 μM) upon reaction with Tz **1** (100 μM) in PBS at 37 °C (n = 3 independent experiments). Relative quantification was done using extracted chromatograms (254 nm, left). In addition, released **CA4** was quantified *via* external calibration (right) to correct for the different absorption of intermediates/products at 254 nm.

4 Iontronic pump assembly

The devices used in each experiment were expressly designed for each platform and purpose (e.g. Eppendorf tubes). The small parts that make up each device and the apparatus were designed in OnShape, a web-based 3D CAD tool, and they were printed using Form 3B+ (Formlabs, USA), a stereolithography (SLA) 3D printer, with high temperature and clear resins depending on the purpose of prints. Building of devices was delicately carried out under a microscope, and if necessary, the UV curable adhesive (NOA86, Norland Products, Inc, USA) was used. To ensure a firm bond between the small prints after applying the optical adhesive, all parts were placed in a UV oven, which was set to 60 °C while irradiating with 405 nm UV light for 12-18 h. Before and after the installation of the capillary into the assembled 3D-printed structure, special care was taken to continuously expose it to 0.1 M KCl or 0.1 M NaCl to maintain conditions and performance of the ion exchange membrane as it was manufactured.

Two different electrode materials (Ag/AgCl and Pt) were used in this research. For the platinum electrode fabrication, which was usually implemented as a working electrode and/or as counter electrode, platinum was sputtered (100 W RF power, 3.9 mTorr process pressure, 7.6 sccm Ar) onto polyimide film cleaned with acetone and isopropanol. The film deposited with platinum was cut into the shapes of the working and counter electrodes using a laser cutting machine (MetaQuip B.V., Netherlands) equipped with a 355 nm pulsed UV laser. To remove debris and ashes that formed during the cutting process that could adversely affect the measurement results, the cut electrode was thoroughly cleaned in an ultrasonication bath with acetone and isopropanol and physically wiped with a cleanroom wipe. To prepare the Ag/AgCl electrode, silver wire (World Precision Instruments, LLC, USA) was cut to an appropriate length (e.g., 50 mm). It was subsequently chlorinated (i.e., the surface converted to AgCl) by immersion in NaOCl (3-6%) for 10 min until the color turned light grey and then rinsed with water.

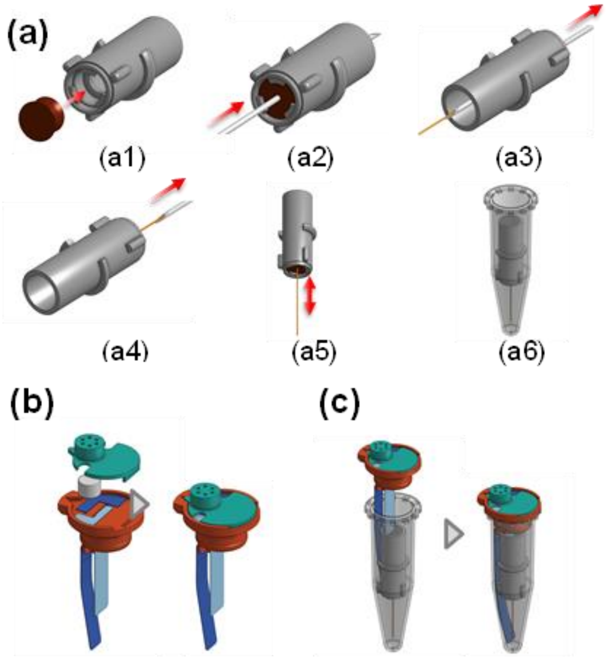

Fig. S3 Assembly process of capillary organic electronic ion pumps (OEIP) for delivery in Eppendorf tubes.

To enable easy and rapid collection of small volume samples (i.e., target solution containing Tz **1**) transported by capillary IPs, the cylindrical device was designed to fit 0.5 mL Eppendorf tubes, that are commonly used in the laboratory, allowing the tubes to be tightly sealed by mounting the device itself. **Fig. S3** shows the device assembly process. The rubber septum of which one side is covered with polytetrafluoroethylene was inserted into the designated port (**Fig. S3a1**). The septum in a circular shape of 3 mm diameter was prepared by cutting it with a biopsy punch on a cutting pad. By using a syringe needle (26 gauge) as guiding tube for the capillary, the syringe was inserted into the septum along the central axis of the 3D-printed barrel-shaped reservoir (**Fig. S3a2**). The capillary was subsequently pushed through the

needle so that the end of the capillary was positioned 3-5 mm from its end (**Fig. S3a3**). The capillary was secured in the septum by carefully removing the syringe (**Fig. S3a4**). At this point, if needed, the length of the capillary exposed to the outside of the reservoir part could be manually adjusted (**Fig. S3a5**) so that the tip was not physically in contact with the inner wall of the microtube after insertion (**Fig. S3a6**). The 3D-printed lid intended to seal the Eppendorf tube was built by assembling the working and counter electrodes (bright and dark blue), a magnet (ϕ 3 x 2 mm, grey), and a 3D-printed holder (red and turquoise) (**Fig. S3b**). To prevent evaporation during measurements, all components of the lid were secured using UV curable adhesive. Following assembly, the source and target reservoirs were filled with their respective electrolyte solutions: source reservoir with 5 mM **1** (50-80 µL) and target reservoir with PBS (50-100 µL). Sealing of the Eppendorf tube with the integrated lid immersed both working and counter electrodes in the solutions, completing device preparation for subsequent pumping experiments (**Fig. S3c**).

5 Iontronic delivery

5.1 Instruments and materials

The ion pumps (IPs) were operated under constant current conditions using an 8-channel OctoStat30 potentiostat (Ivium Technologies), interfaced with the target and source electrodes *via* custom-fabricated pinhole connectors, enabling direct control and monitoring of the IPs (see **Fig. S4**).

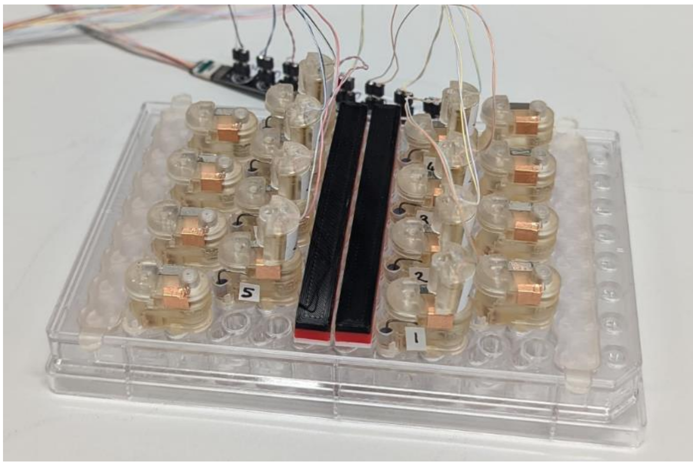

Fig. S4 Set-up of 16 iontronic devices on a transwell 96-well microplate. Eight devices are connected to the OctoStat30 potentiostat *via* a custom-fabricated 8-channel pinhole connector. The remaining eight devices are sealed with PDMS stoppers to minimize evaporation of the source solution and remain inactive.

The concentration of delivered **1** in the target solution was measured using a fluorogenic assay *via* the C2R of 4-methylumbelliferone (**4MU**) from rTCO-DMEDA-4MU (**2**). Fluorescence measurements were performed on a plate reader (Synergy H1 microplate reader, BioTek) at 25 °C using 384-well microplates (Greiner Bio-One, Polypropylene, black, non-sterile, Flat bottom, 82051-318, VWR), which were covered (Nunc™ Sealing Foil, cat. no. 232702, Thermo Scientific) to prevent evaporation during measurements.

5.2 Iontronic delivery in constant-bias mode

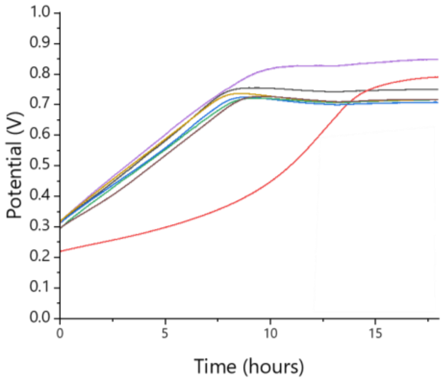

Fig. S5 Potential-time traces for **loading** of 8 devices at +20 nA used to collect the data depicted in **Fig. 2c**. Ion exchange from K^+ to **1** is indicated by a plateau in potential, occurring at around 8 h. One device exhibited an extended loading phase of 15 h (red curve), but reached a comparable potential plateau and was therefore included in the data set.

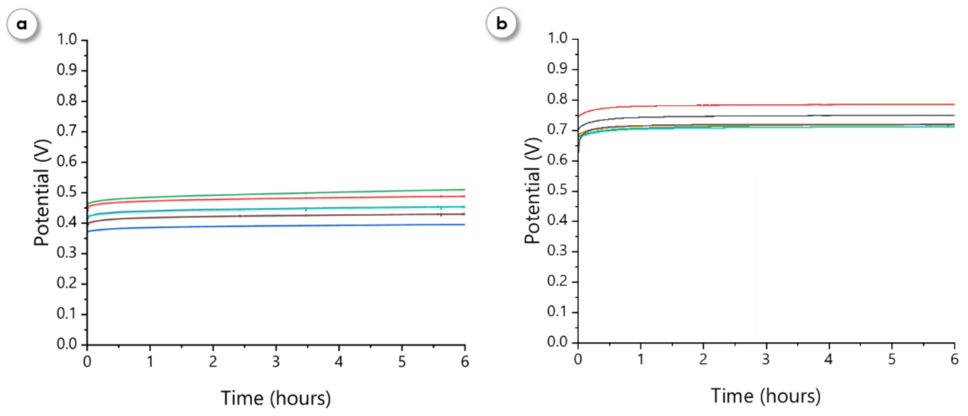

Fig. S6 Potential-time traces of 8 devices for **active delivery** used to collect the data depicted in **Fig. 2c** using Ag/AgCl electrodes. **a**, Applied current **+10 nA** and **b**, Applied current **+20 nA**.

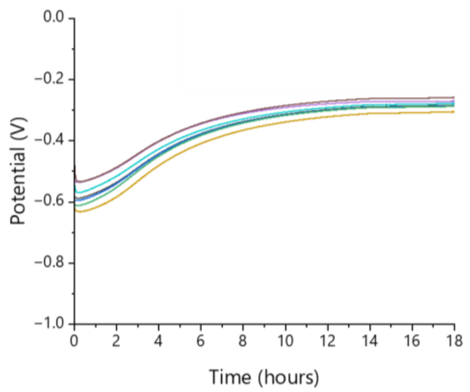

Fig. S7 Potential-time traces of 8 devices used for **reverse operation** using Ag/AgCl electrodes. Applied current **-20 nA**.

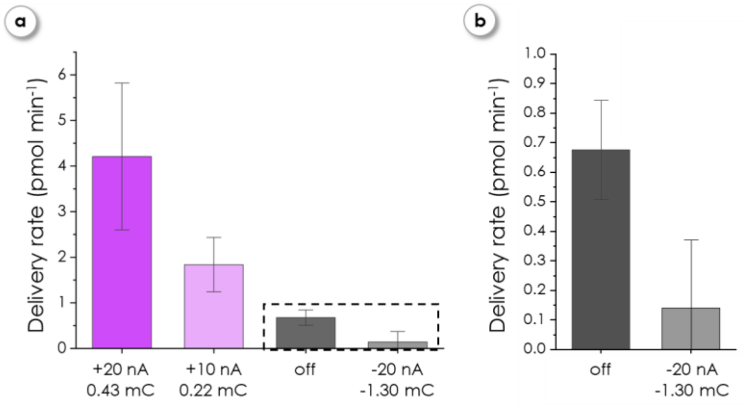

Fig. S8 a, Delivery rates of **1** with varied applied current. Applying a reverse bias greatly reduces the delivery rate compared to the “off” state and thus was used for further experiments. Delivery rates for +20 nA and +10 nA were determined after 6 h of ion pump operation, “off” and -20 nA reverse rates were determined after 18 h. A floating voltage was used in the “off”-case. **b**, Magnified view of the dashed box in (**a**) showing delivery rates for “off” and -20 nA. Bars represent mean ± SD; points represent individual independent experiments (n = 6-8).

5.3 Iontronic delivery in step-function mode

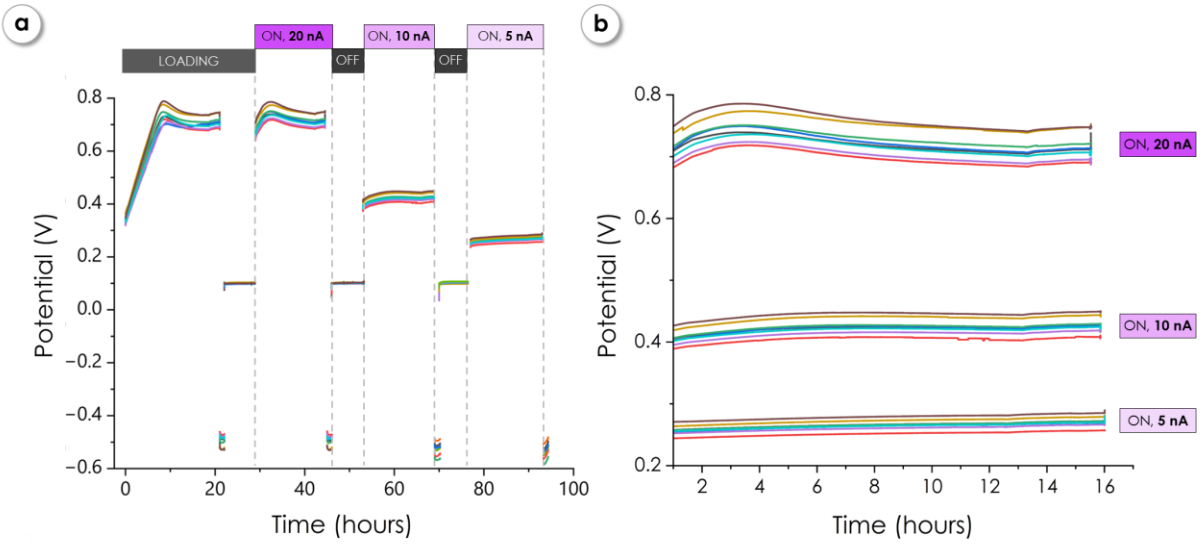

Fig. S9 Potential-time traces of 8 devices in step-function mode. **a**, Full overview of step-function mode. **b**, Magnified view of "ON" potential traces, showing full functionality of all devices as indicated by stable potential-time traces and the scalability of potential with respect to the applied current (decreasing from +20 nA to +10 nA to +5 nA from top to bottom).

6 Iontronic release of CA4 from 4 on cells

6.1 CA4 release after iontronic Tz delivery

Iontronic devices (AMPS/PEGDA 85/15 mol% ratio at 25 wt% in deionized water, 15 mm length, 25 µm inner diameter, 150 µm outer diameter; source reservoir concentration: 5 mM **1**) were installed on 0.5 mL Eppendorf tubes using the custom 3D-printed housings. Ion pumps were assembled as described in **Fig. S3**. The channel loading was done at +20 nA over 10 h into 50 µL PBS. After reaching a steady potential indicating a fully occupied CEM, the target solution was exchanged with 50 µL sulfo-cTCO-DMEDA-CA4 (**4**) in PBS (50 µM, containing 10% DMSO) and run at +20 nA for different time periods (3 h, 6 h, and 9 h). Additionally, delivery of K^+ (0.1 M KCl in the source reservoir) was performed for 9 h instead of **1**. After a 24 h incubation period (including delivery time), all samples were subjected to HPLC analysis. PDA data were collected for all samples. Relative quantification of intermediates and products was done using extracted chromatograms (wavelength: 254 nm). The measurements were conducted in triplicates. Delivery of K^+ demonstrated prodrug **4** stability under electrochemical conditions showing no release of **CA4** (**Fig. S10a**). In contrast, iontronic transport of Tz **1** (5.0 ± 0.8 pmol min^{-1}) confirmed excellent temporal control of **CA4** release (**Fig. S10b**).

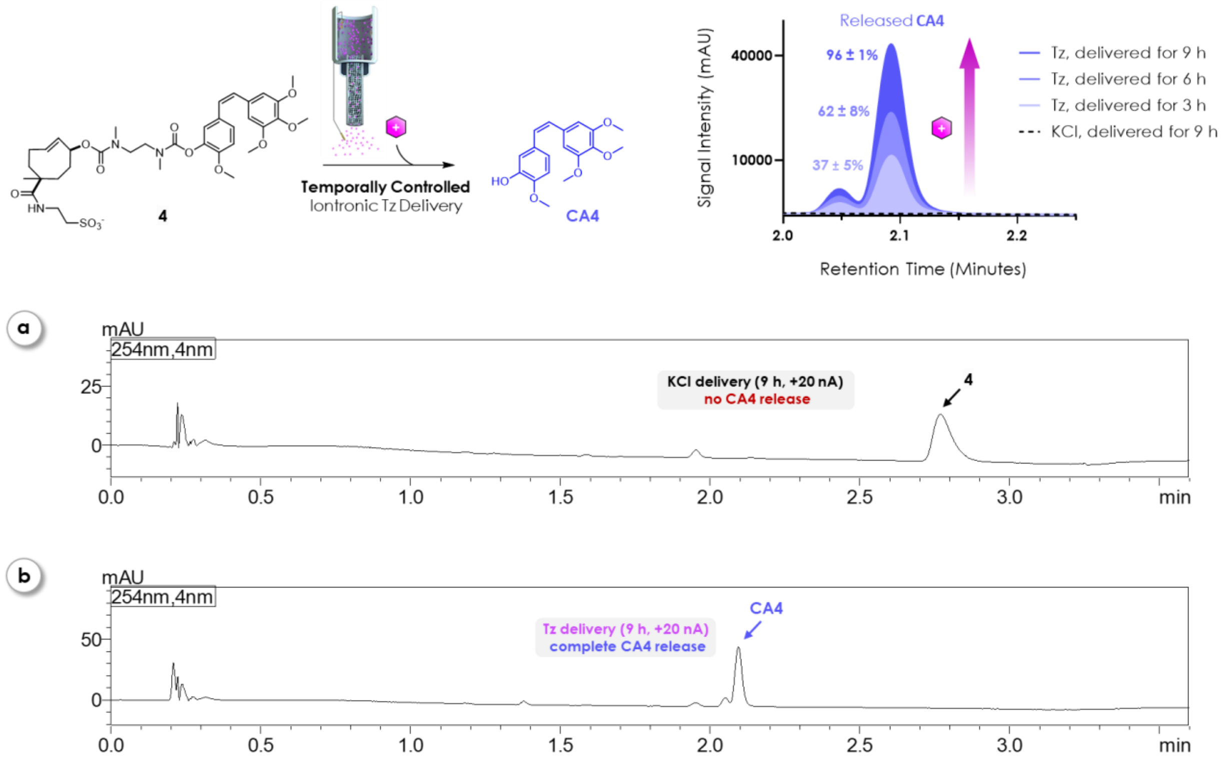

Fig. S10 HPLC chromatograms (254 nm) of target solutions containing prodrug **4** (50 µM) under different pumping conditions after a 24 h incubation period (including delivery times, n = 3): **a**, Iontronic KCl delivery (9 h, +20 nA) confirming the stability of **4** under iontronic delivery conditions; **b**, Iontronic Tz **1** delivery (9 h, +20 nA) showing complete release of **CA4**. Two distinct peaks were observed, consistent with literature reports of partial **CA4** isomerization, and are attributed to **CA4** and its corresponding trans isomer.[6] Both peaks were integrated and used for determination of the total released amount.

6.2 CA4 efficacy

The maximum drug effect was achieved at concentration >100 nM released **CA4** (**4** co-incubated with 5 μM **1**) that resulted in 22% cell viability. At this point, further increases in drug concentration would not lead to any more cell death. The calculated efficacy is the inverse representation of the observed cell viability normalized to the minimal possible cell viability:

$$Efficacy\ (\%) = \left(\frac{100 - observed\ cell\ viability}{100 - minimal\ possible\ cell\ viability}\right) * 100$$

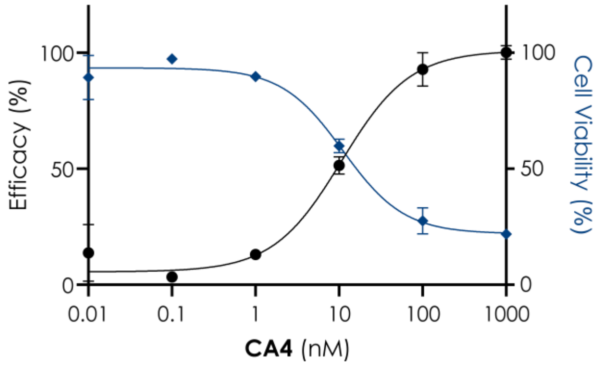

Fig. S11 Relation of cell viability and drug efficacy. Cell viability (blue) decreases with increasing concentrations of released **CA4** (**4** incubated with 5 μM **1)**, and plateaus at a minimum of 22%. Efficacy (black) was calculated based on the formula shown above. Data are shown as mean ± SD; n = 3 independent experiments.

6.3 Potential-time traces

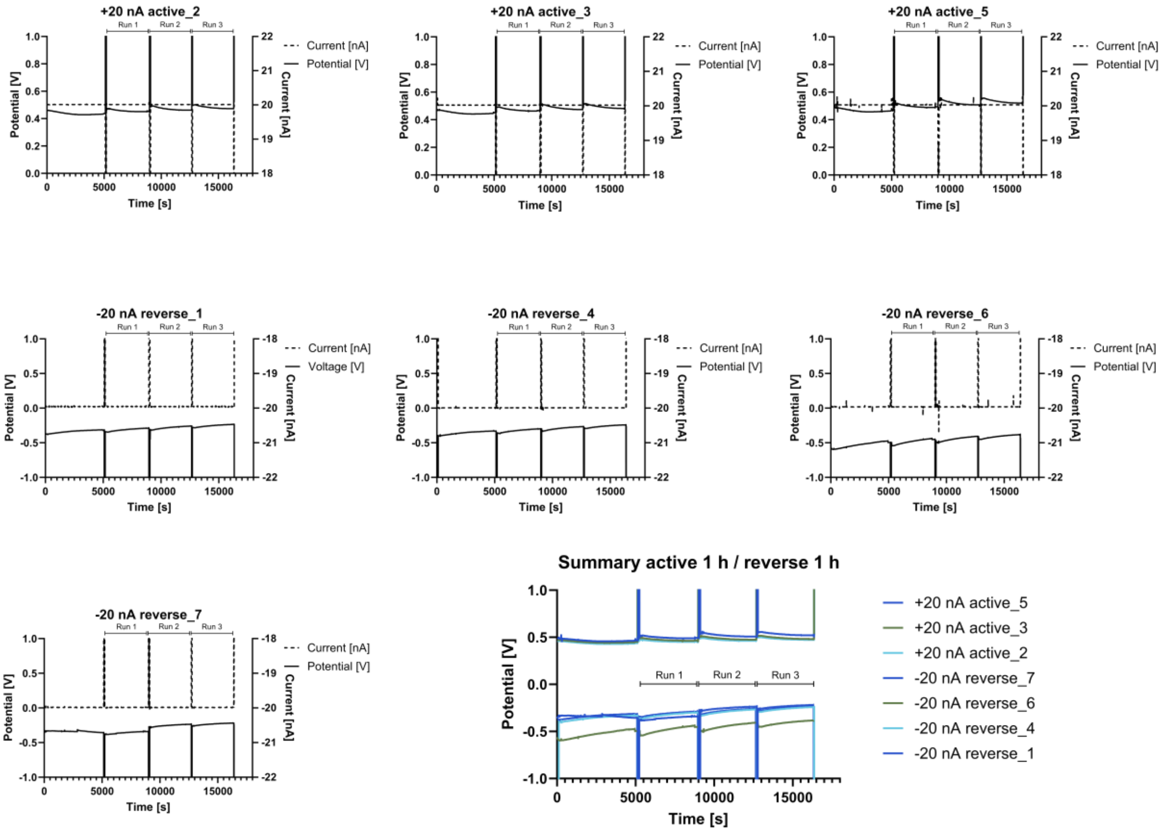

Fig. S12 Potential-time traces and overlay of loading and delivery of **1** for three devices, each operated for 1 h at +20 nA and 4 devices operated in reverse for 1 h at -20 nA. Each run was repeated 3 times without stopping the power supply while changing well plates. Voltage peaks between the runs indicate short electronic contact loss between disconnecting and reconnecting of the electrodes.

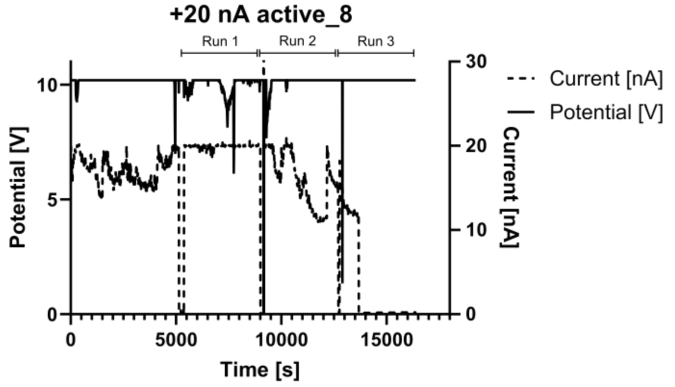

Fig. S13 Exemplary voltage trace of one faulty device (device "8"). The corresponding cell viability data was excluded due to reaching the maximum voltage of 10 V, indicating non-functional Tz delivery.

7 Iontronic release of CA4 from 6 on beads

Potential-time traces

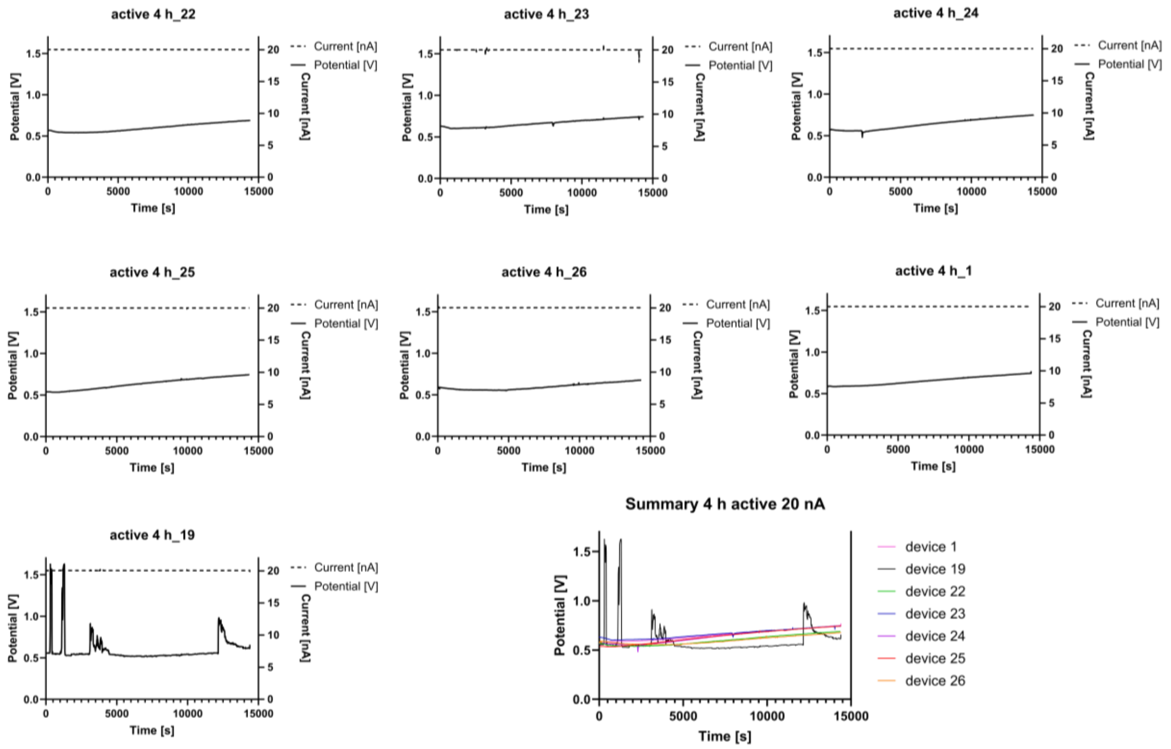

Fig. S14 Potential-time traces and overlay of 7 devices operated for 4 h at +20 nA for the delivery of **1** into wells containing **CA4**-attached beads ("4 h active").

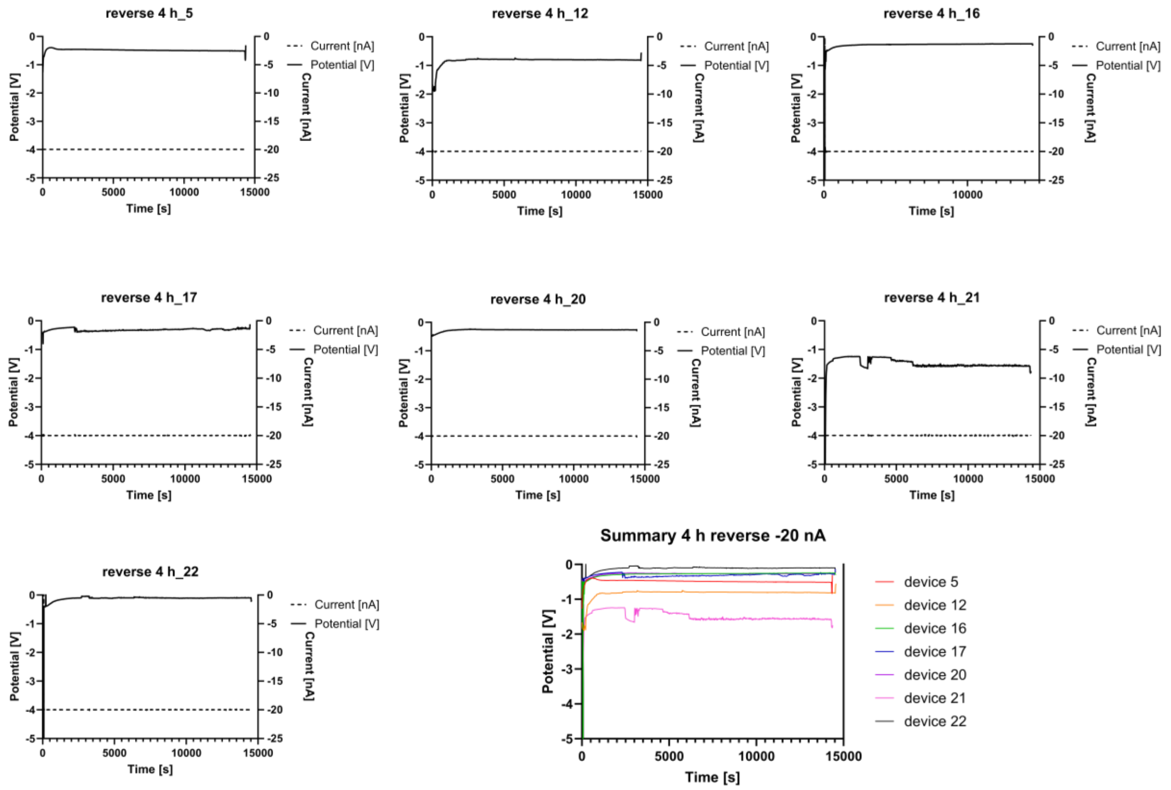

Fig. S15 Potential-time traces and overlay of 7 devices operated for 4 h at -20 nA ("4 h reverse").

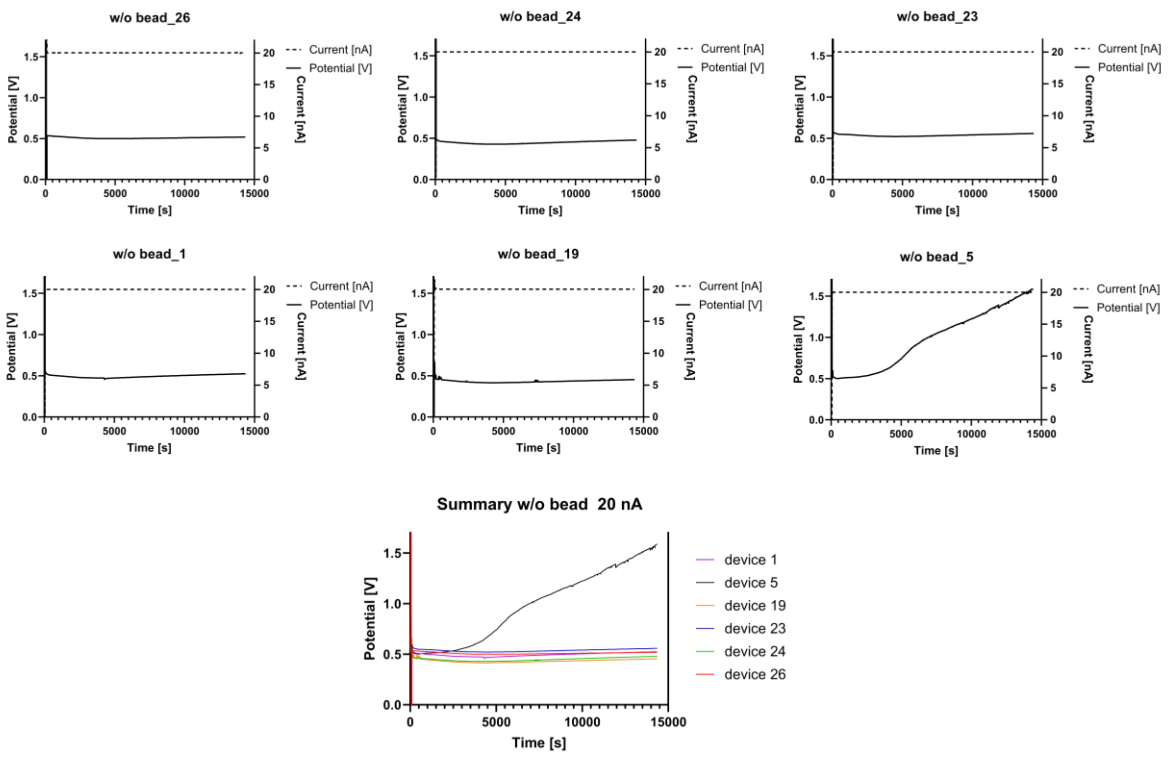

Fig. S16 Potential-time traces and overlay of 6 devices operated for 4 h at +20 nA for the delivery of **1** into wells without beads (“w/o beads”).

8 Iontronic release of BSA from beads

8.1 Potential-time traces

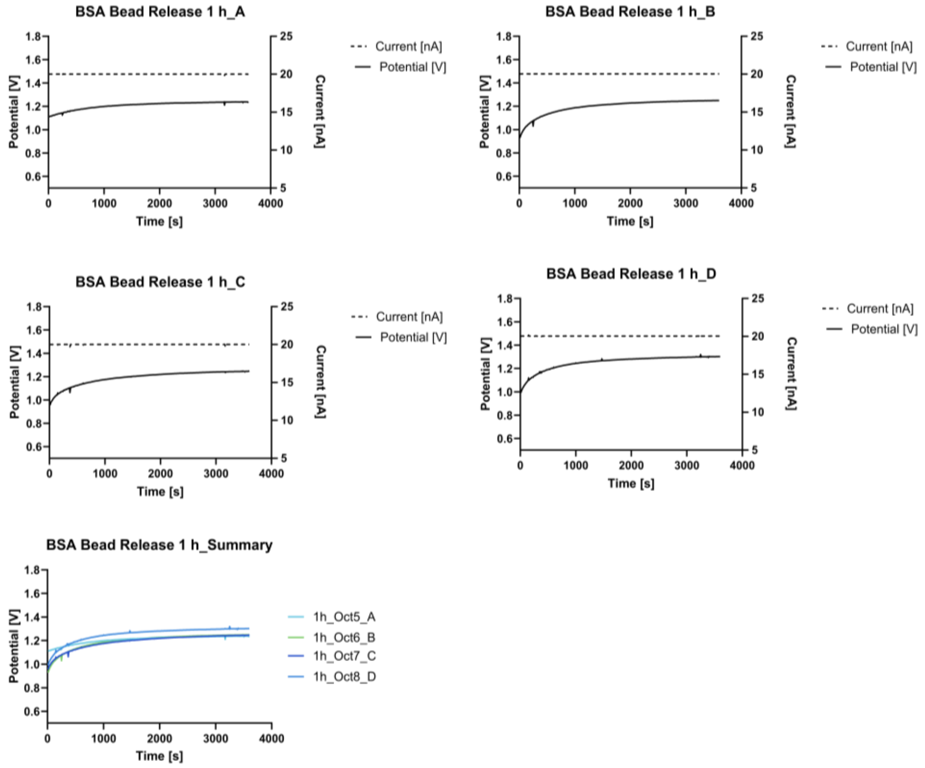

Fig. S17 Potential-time traces and overlay of 4 devices operated for 1 h at +20 nA.

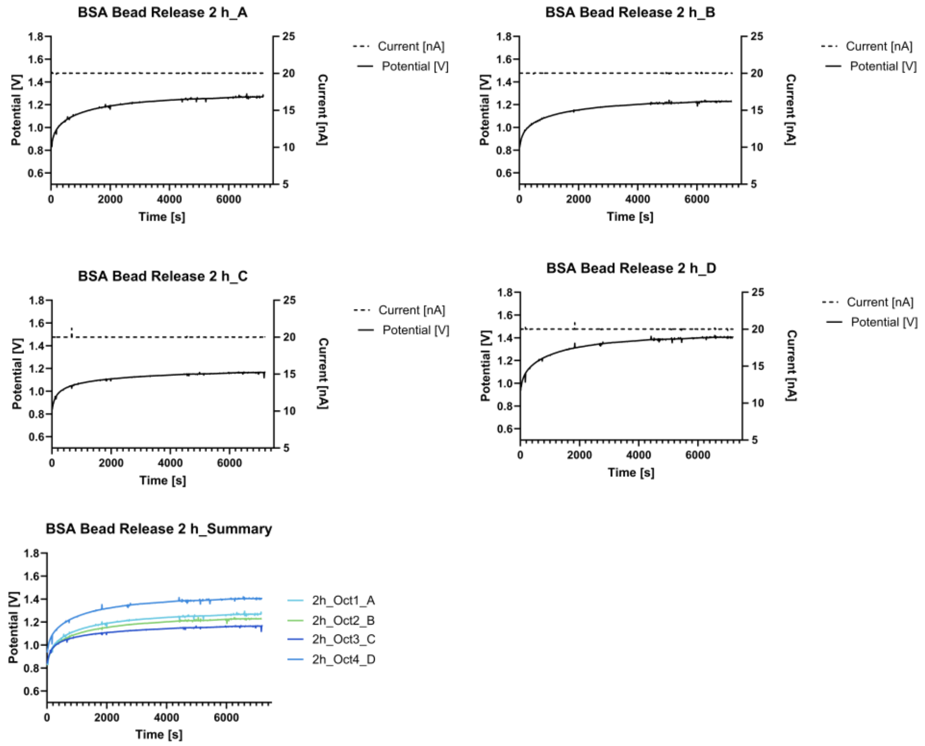

Fig. S18 Potential-time traces and overlay of 4 devices operated for 2 h at +20 nA.

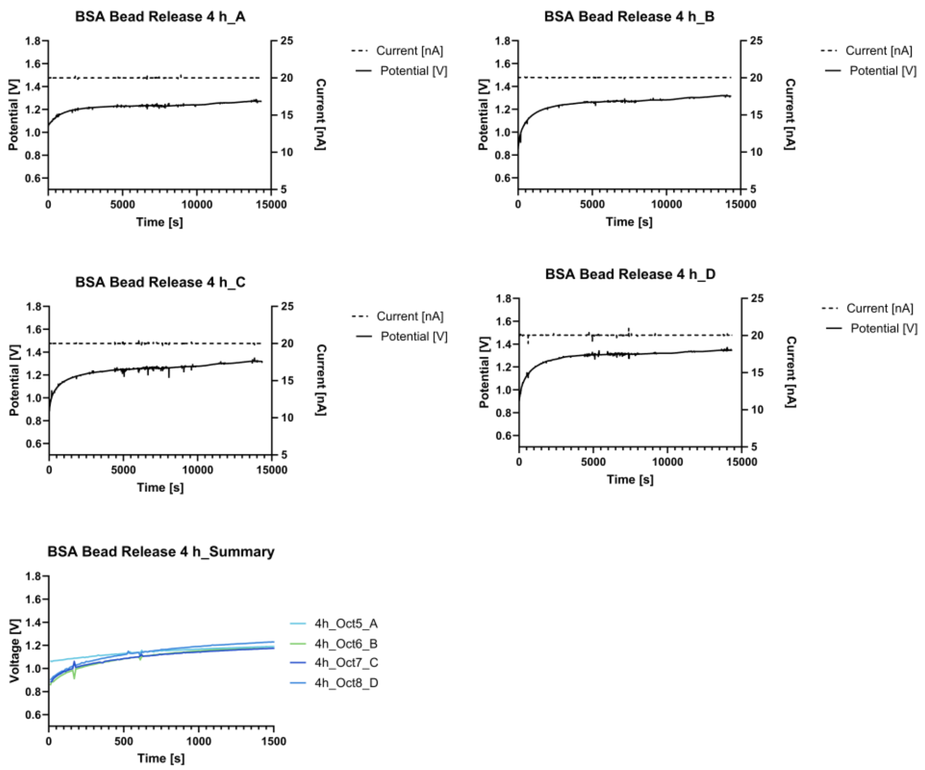

Fig. S19 Potential-time traces and overlay of 4 devices operated for 4 h at +20 nA.

8.2 SDS-PAGE

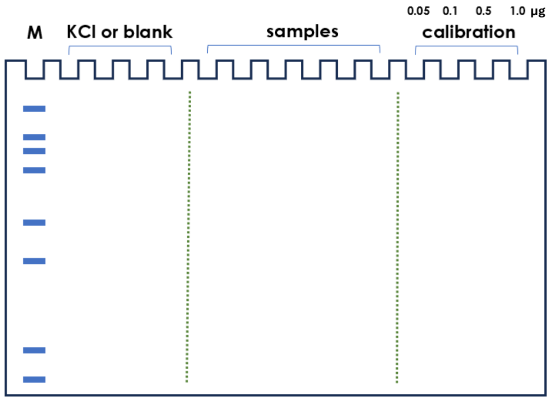

Fig. S20 SDS-PAGE gel loading scheme for protein analysis. The gel lanes were loaded as follows: Lane M, molecular weight marker (M - Bio-Rad – Precision Plus Protein™ All Blue Standards #1610373); first set of 4 lanes, KCl-treated or blank control samples; middle set of 6 lanes, experimental samples; final set of 4 lanes, calibration standards with known **BSA** (as BSA-N_3) amounts (0.05-1.0 µg). The schematic illustrates the relative positions of each group of samples within the gel, with dashed lines indicating the boundaries between sample sets. This loading strategy enables direct comparison of released protein amounts against calibration standards for quantification.

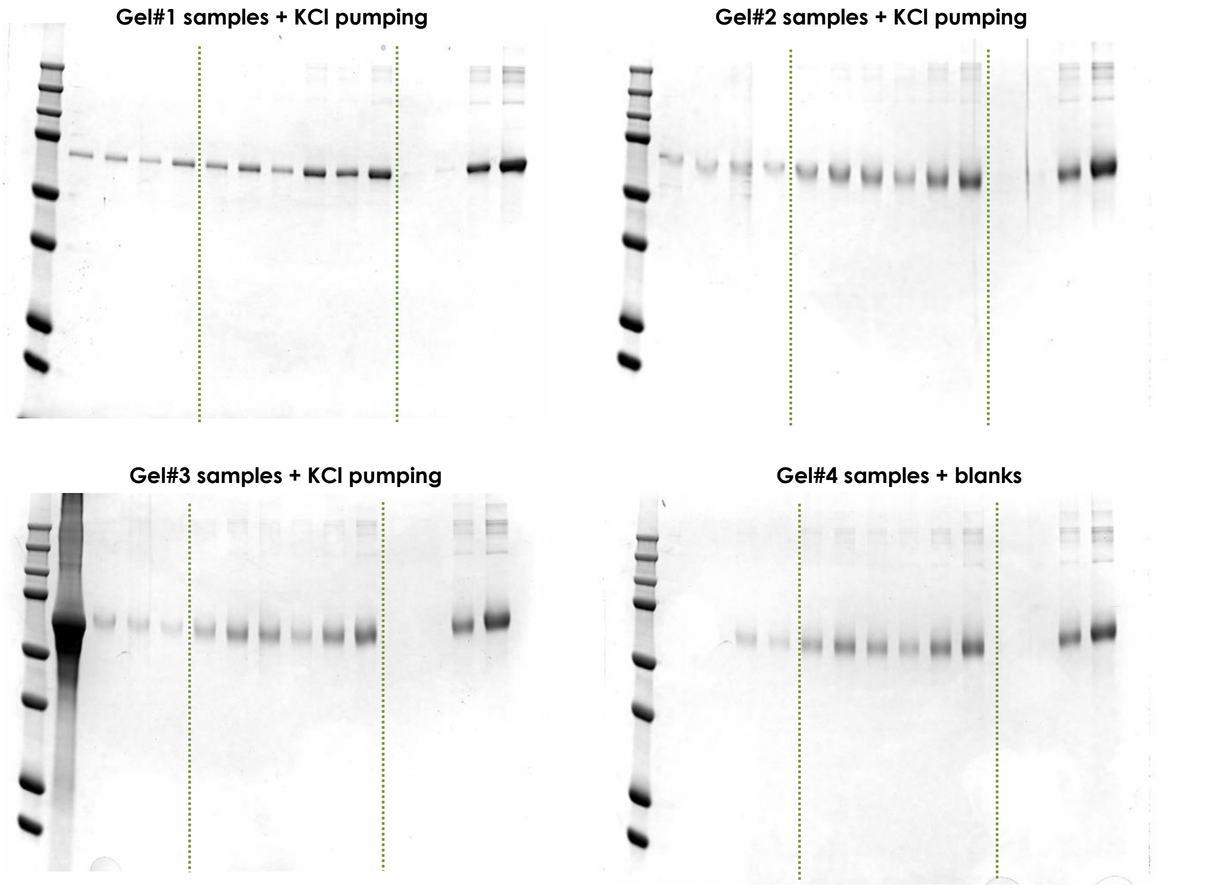

Fig. S21 SDS-PAGE analysis after Coomassie staining of iontronically pumped samples including KCl-pumping and blank control samples across four independent gels (n = 4). Each gel was loaded according to the scheme described in **Fig. S20**: lanes contain molecular weight markers (leftmost), KCl-treated or blank samples (first group), experimental samples (middle group), and calibration standards (right group, with **BSA** concentrations 0.05–1.0 µg). Green dashed lines visually guide boundaries between sample sets.

8.3 Background BSA Release Observed During K^+ Delivery

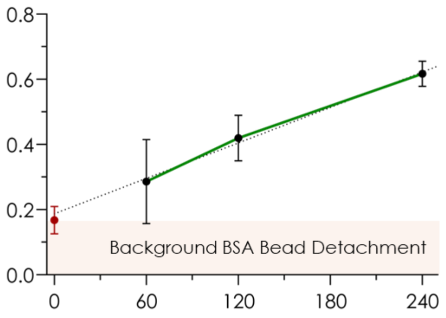

Fig. S22 Iontronic K^+ delivery (0.1 M KCl, 4 h, +20 nA) used as a control resulted in background **BSA** signal, attributed to passive protein dissociation or electrically induced bead detachment (red data point). This data was used to subtract the background signal in Fig. 5b. Data are shown as mean ± SD of n = 4 independent experiments per time point.

9 Cyclic voltammetry (CV)

9.1 Instruments and sample preparation

Electrochemical measurements were performed using an Autolab potentiostat (PGSTAT101) in combination with Nova software and a three-electrode setup consisting of an Ossila platinum working electrode (283 mm^2), an Ossila platinum wire counter electrode (37 mm × 0.5 mm), and an Ossila Ag/AgCl reference electrode (35 mm × 0.5 mm) in contact with an aqueous 100 mM KCl solution. The stock solutions of Tz **1** (0.5 mM) and KCl (0.1 M) were prepared in distilled water, and the electrolyte solution was degassed with argon for 10 min prior to use.

9.2 CV measurement

The cyclic voltammogram was recorded by sweeping the potential from -0.7 V to +0.4 V, starting at 0 V, at a scan rate of 20 mV/s. All measurements were carried out at room temperature under ambient pressure.

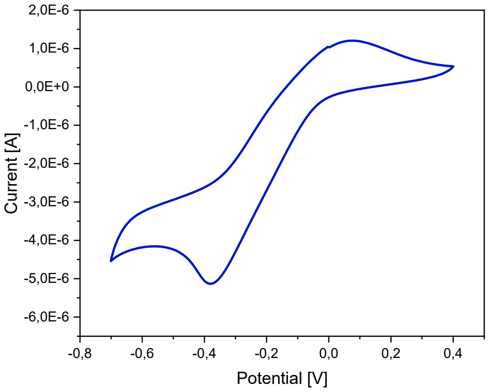

Fig. S23 Cyclic voltammogram of Tz **1** in aqueous solution (0.5 mM, 0.1 M KCl) using a Pt working and counter electrode and an Ag/AgCl reference electrode. The scan was initiated at 0 V and cycled between -0.7 V and +0.4 V at a scan rate of 20 mV/s. A cathodic peak at -0.45 V corresponds to the electrochemical reduction of the tetrazine to its dihydrotetrazine form. The broad peak-to-peak separation and reversible oxidative feature at -0.1 V indicate reversible behavior. These data confirm that **1** is electrochemically stable and reversibly reducible and oxidizable at potentials used in iontronic deliveries.

10 NMR Spectra

Compound **S1**, ^{1}H NMR

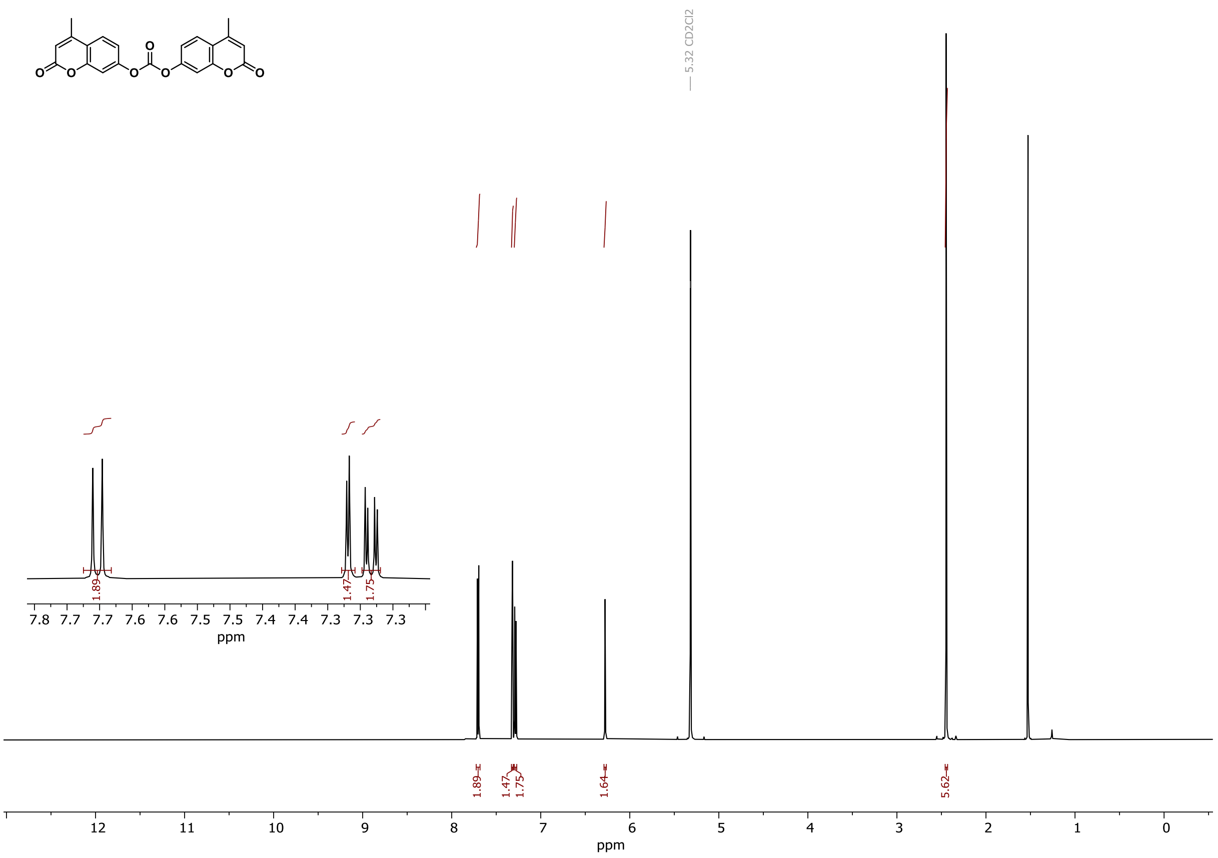

Compound **S1**, ^{13}C NMR

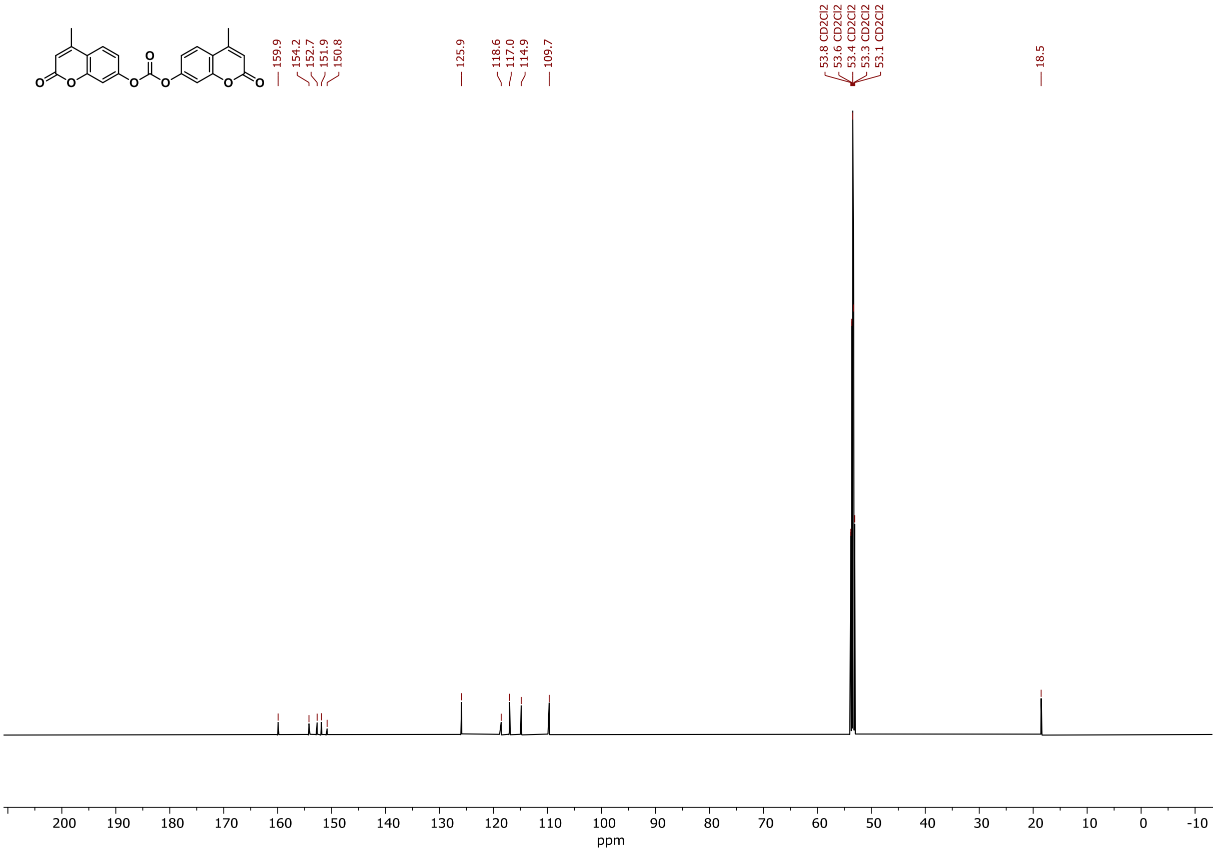

Compound **2**, ^{1}H NMR

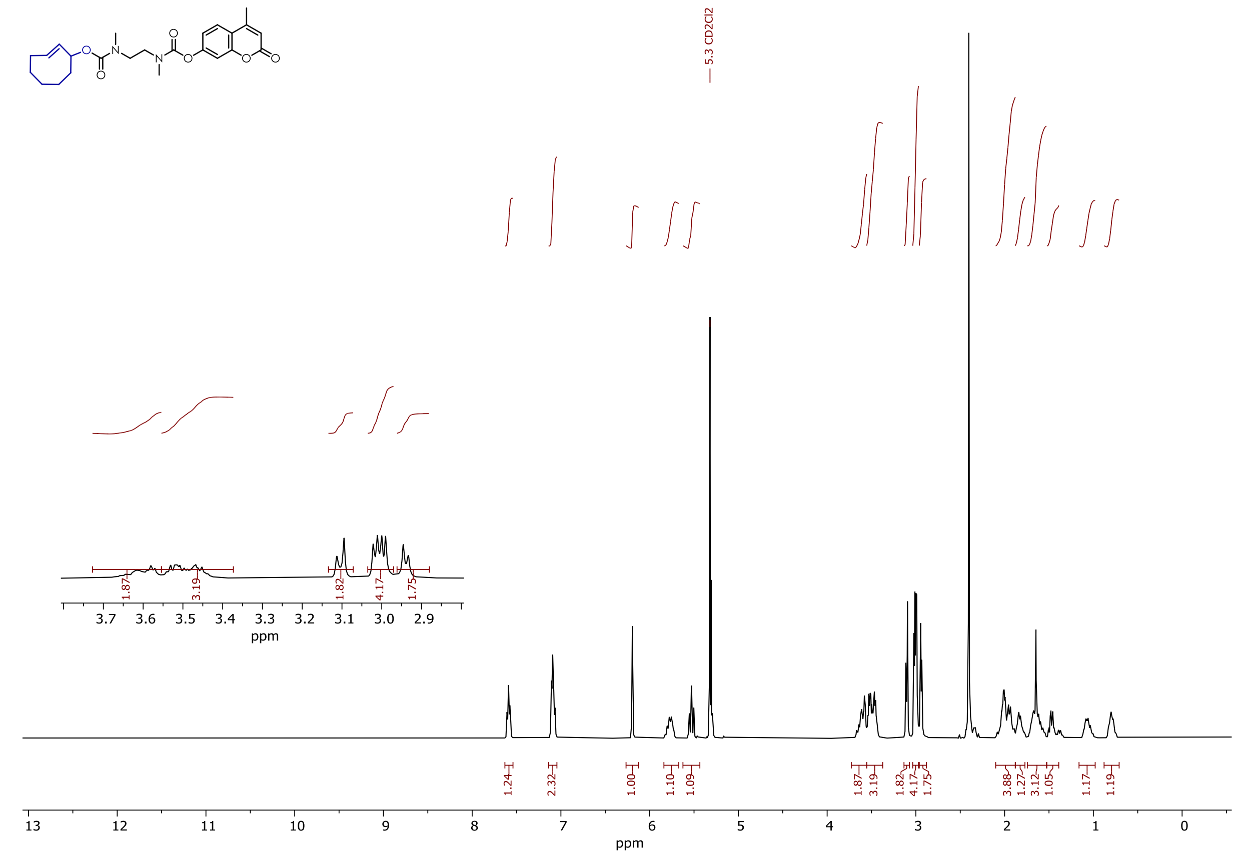

Compound **2**, ^{13}C NMR

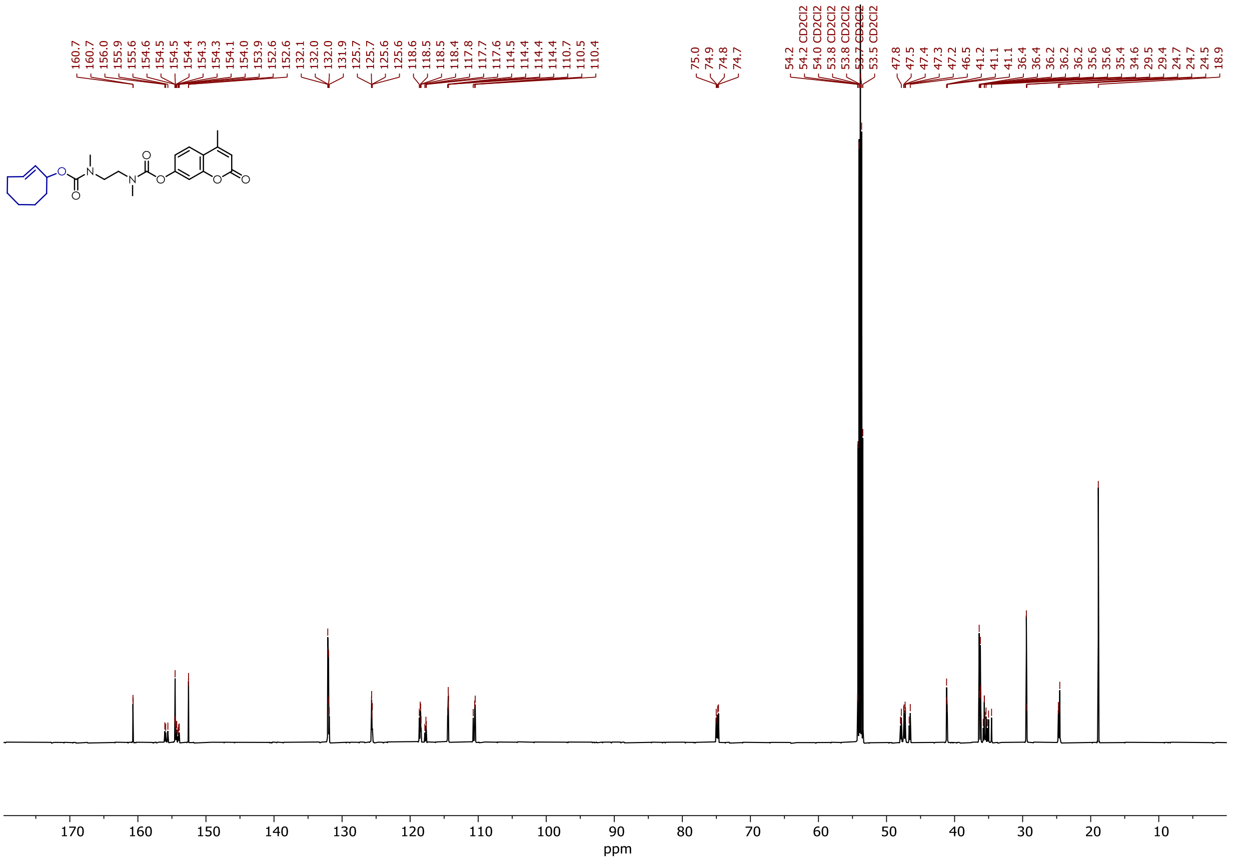

Compound **3**, ^{1}H NMR

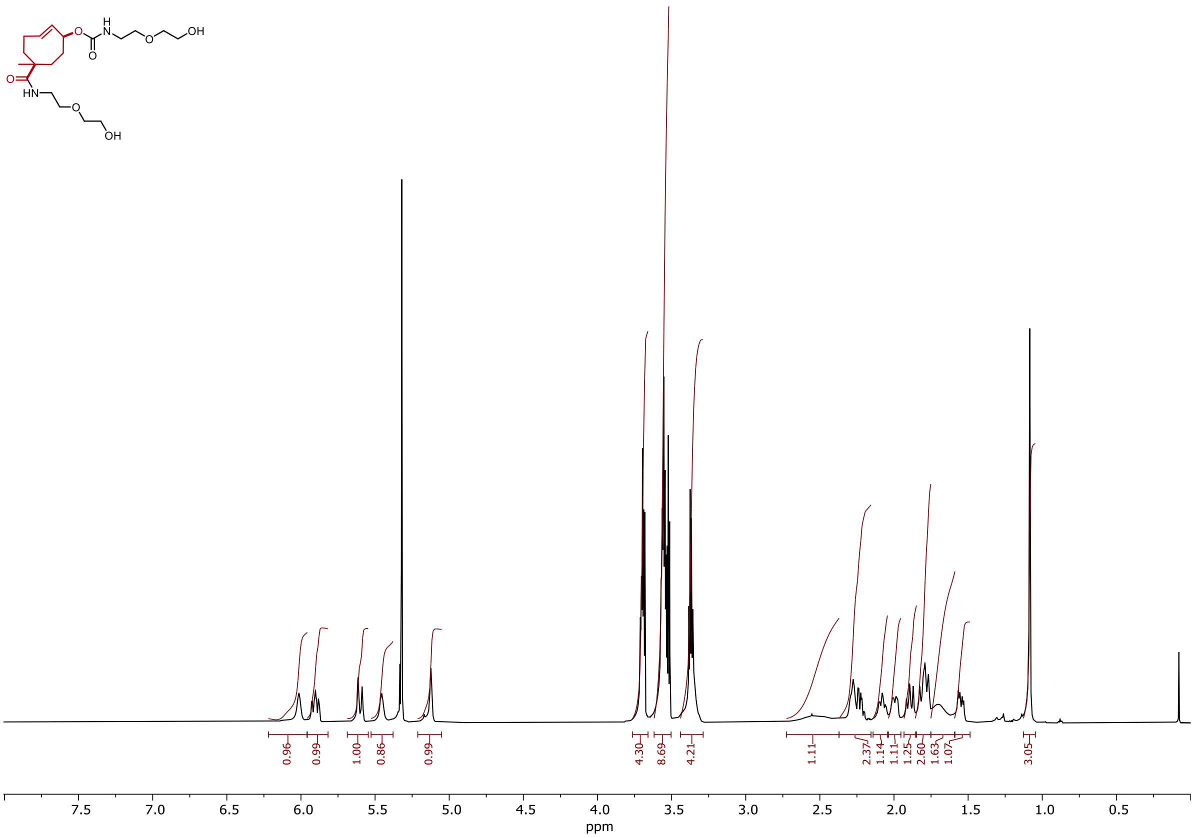

Compound **3**, ^{13}C NMR

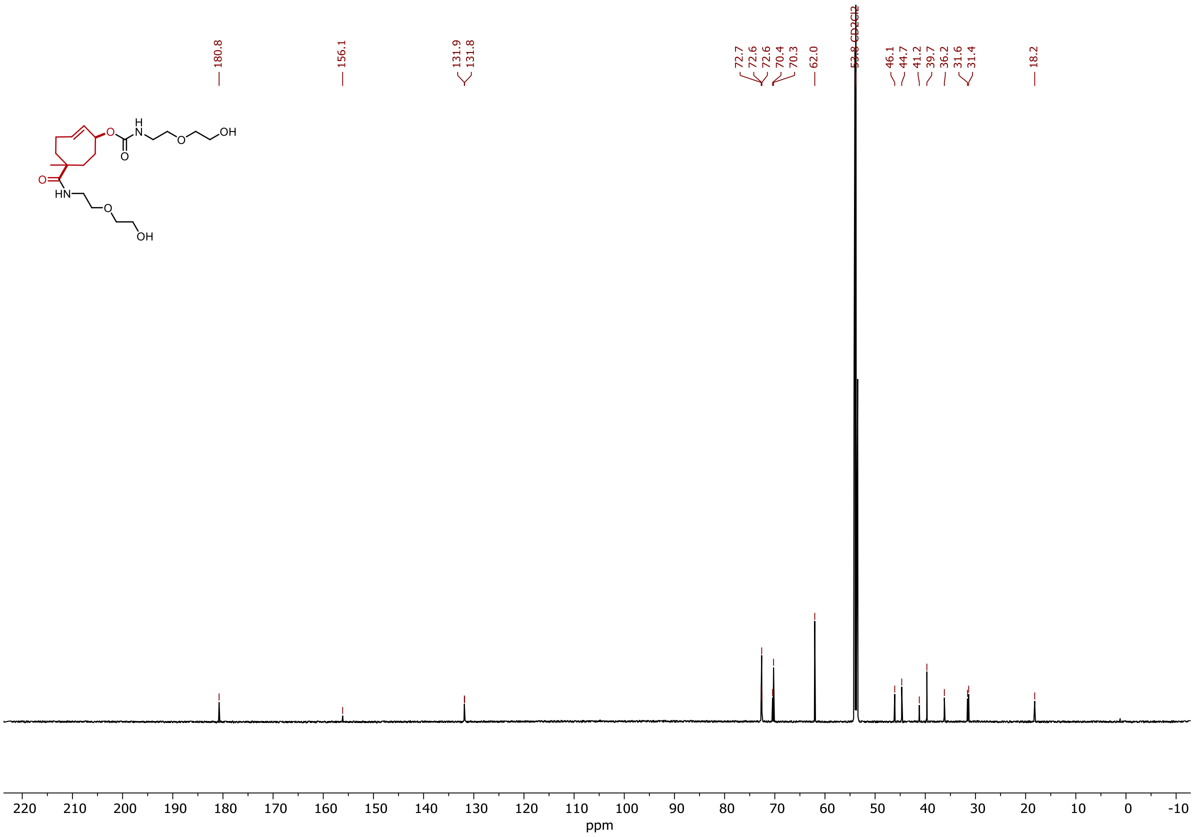

Compound **S2**, ^{1}H NMR

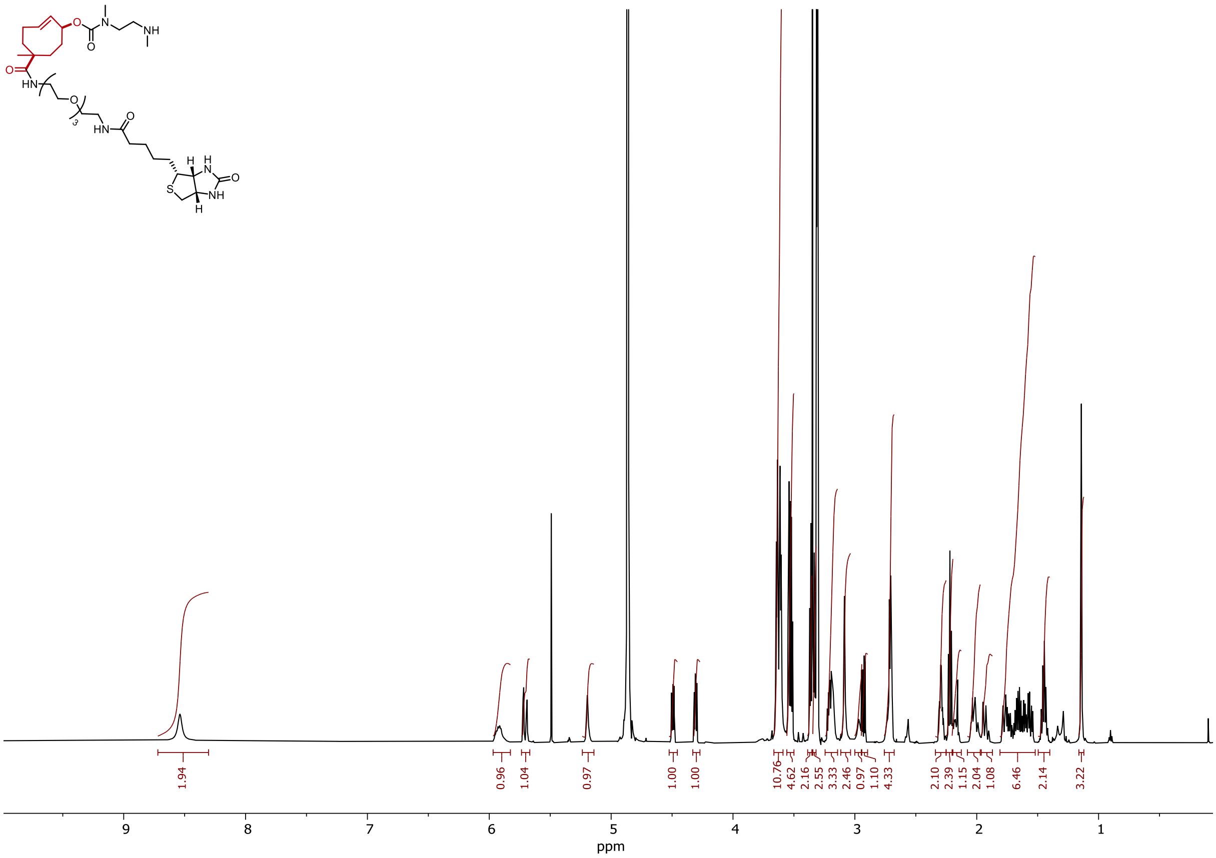

Compound **S2**, ^{13}C NMR

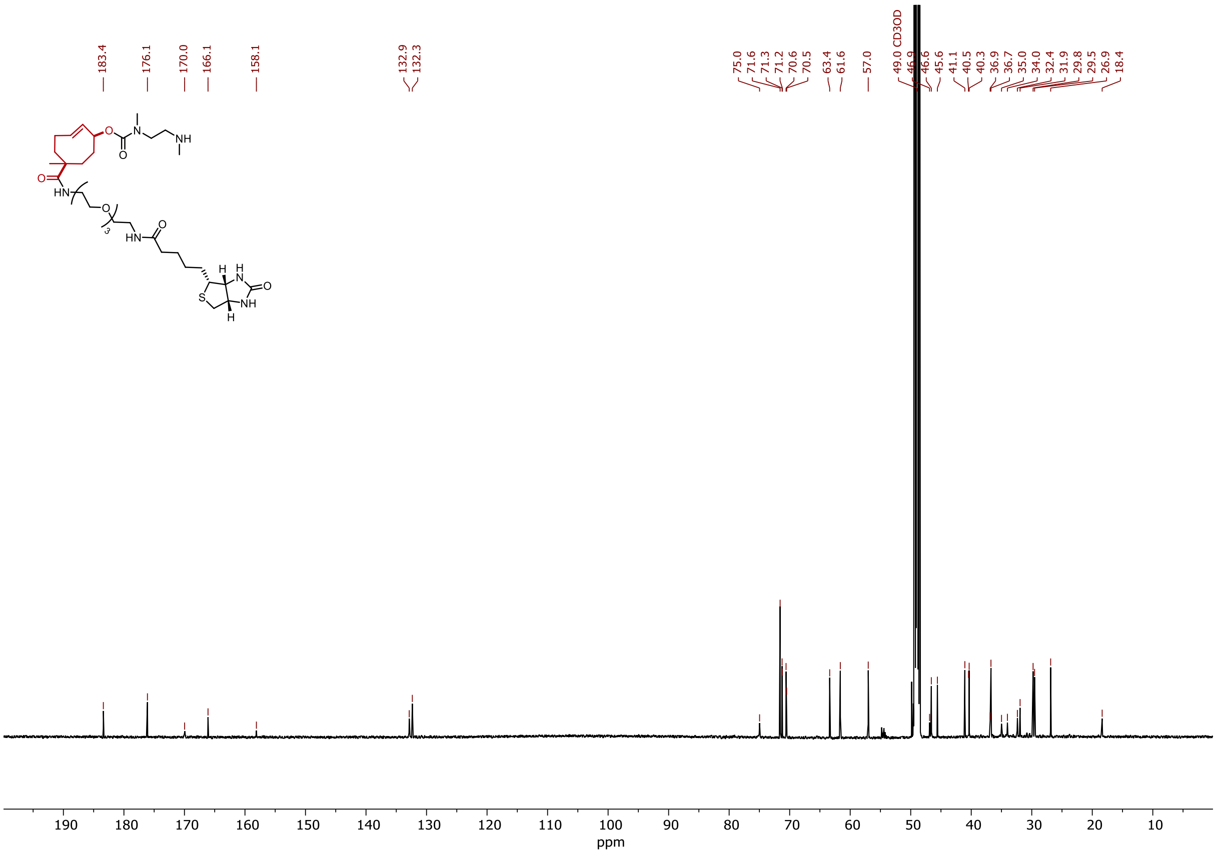

Compound **6**, ^{1}H NMR

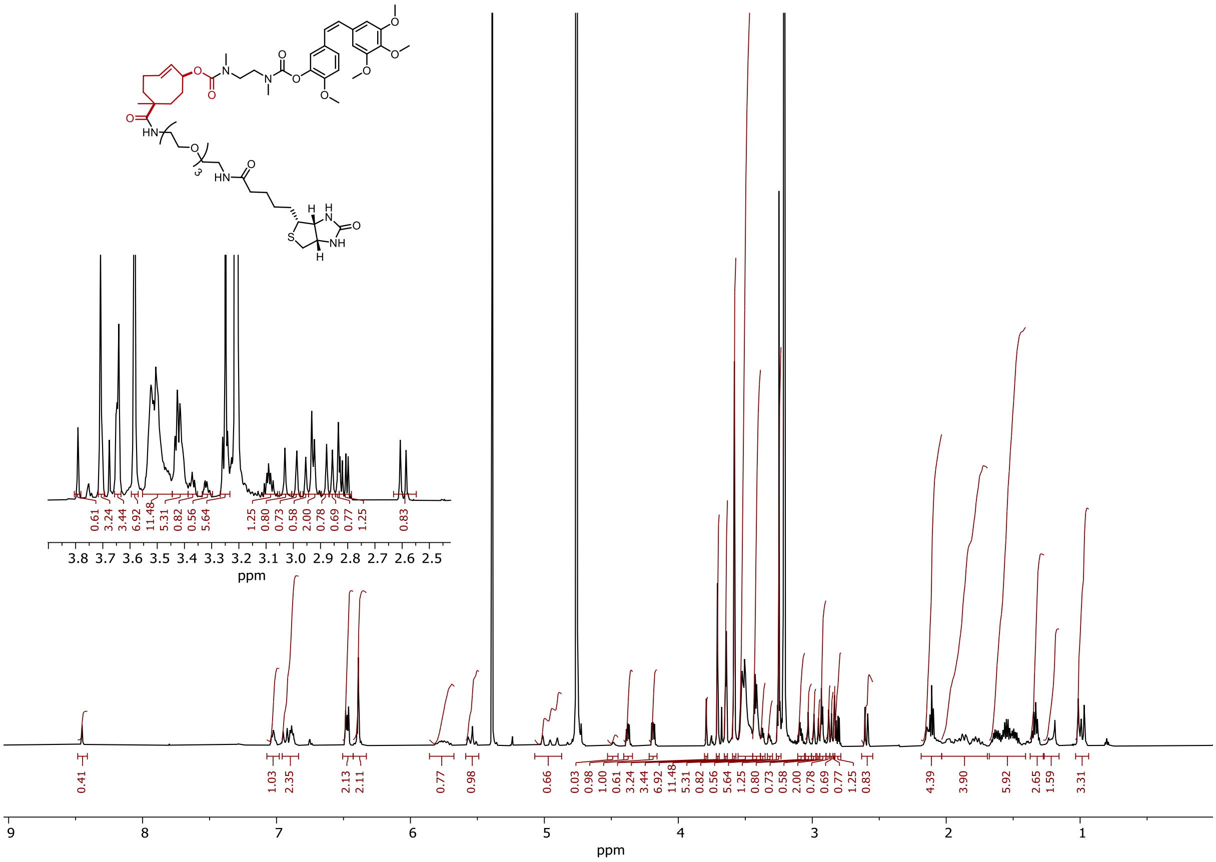

Compound **S3**, ^{1}H NMR

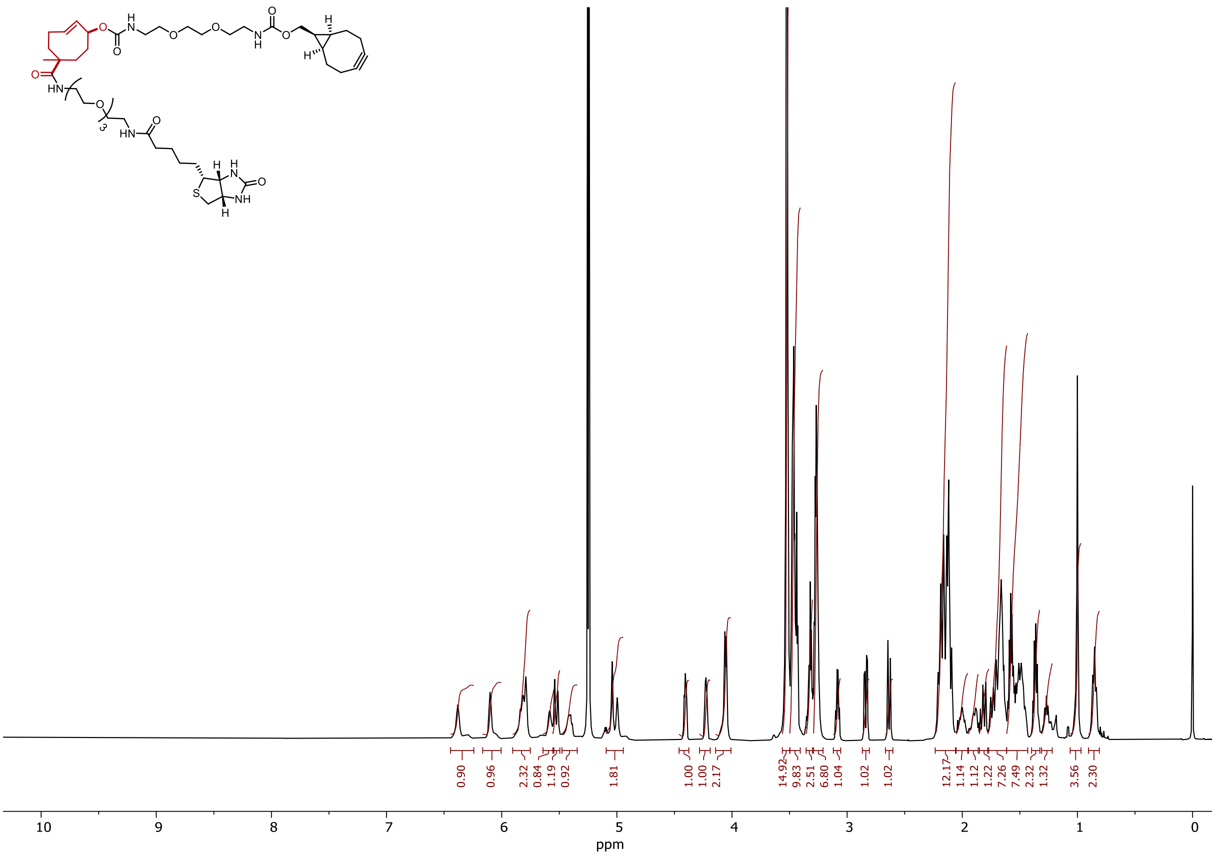

Compound **S3**, ^{13}C NMR

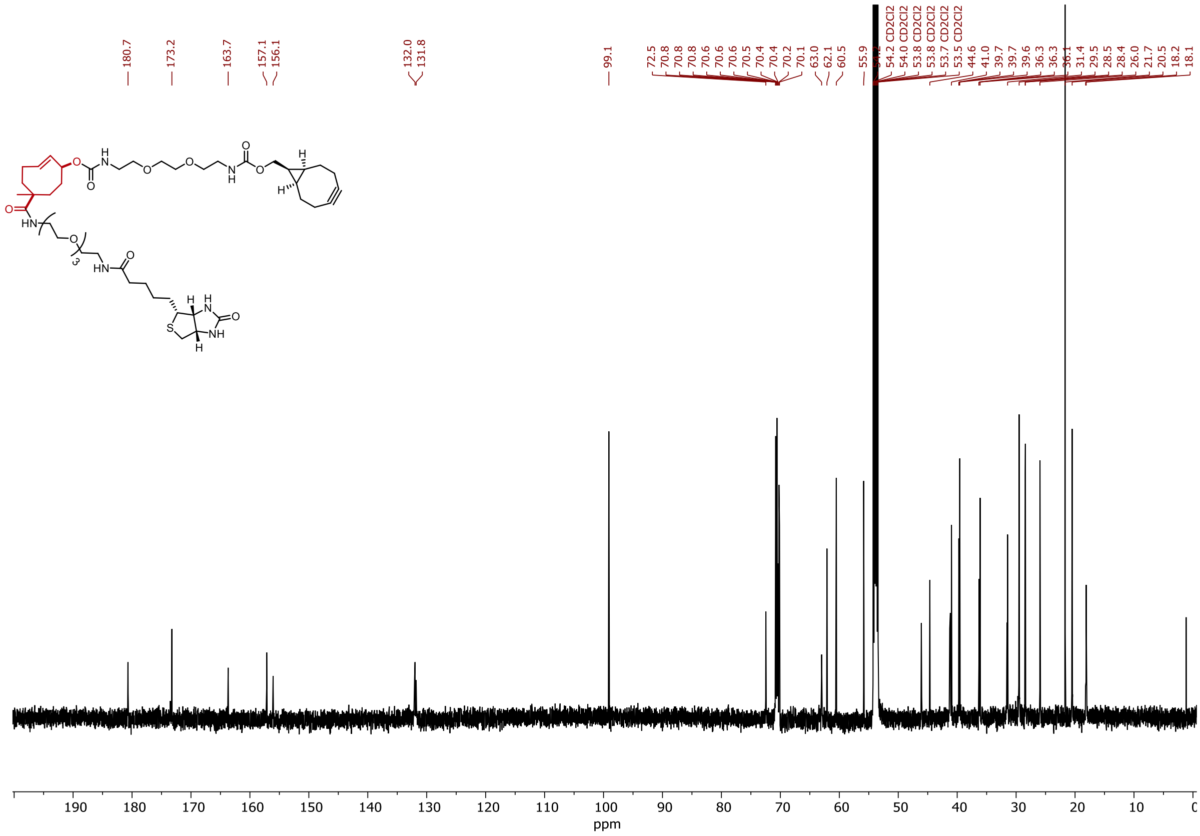

11 References

1 Sarris, A. J. C. *et al.* Fast and pH-Independent Elimination of trans-Cyclooctene by Using Aminoethyl-Functionalized Tetrazines. *Chemistry – A European Journal* **24**, 18075-18081 (2018). https://doi.org/10.1002/chem.201803839

2 Versteegen, R. M., Rossin, R., ten Hoeve, W., Janssen, H. M. & Robillard, M. S. Click to Release: Instantaneous Doxorubicin Elimination upon Tetrazine Ligation. *Angewandte Chemie International Edition* **52**, 14112-14116 (2013). https://doi.org/10.1002/anie.201305969

3 Keppel, P. *et al.* Tetrazine-Triggered Bioorthogonal Cleavage of trans-Cyclooctene-Caged Phenols Using a Minimal Self-Immolative Linker Strategy. *ChemBioChem* **23**, e202200363 (2022). https://doi.org/10.1002/cbic.202200363

4 Rossin, R. *et al.* Triggered Drug Release from an Antibody–Drug Conjugate Using Fast "Click-to-Release" Chemistry in Mice. *Bioconjugate Chemistry* **27**, 1697-1706 (2016). https://doi.org/10.1021/acs.bioconjchem.6b00231

5 Ellison, S. L. R., Rosslein, M., Williams, A. & *Quantifying uncertainty in analytical measurement*. (2000), Second Edition

6 Karatoprak, G. Ş. *et al.* Combretastatins: An Overview of Structure, Probable Mechanisms of Action and Potential Applications. *Molecules* **25**, 2560 (2020). https://doi.org/10.3390/molecules25112560
