# Supplementary material for: Iontronic click-to-release enables electrically controlled delivery of drugs and biomolecules beyond charge and size limitations
Source: Nat Commun. 2026 Mar 31;17:4629. doi: 10.1038/s41467-026-70985-0 (PMC13199424; doi:10.1038/s41467-026-70985-0)
Supplement: Supplementary file 2 — Reporting Summary [file 41467_2026_70985_MOESM2_ESM.pdf]

nature portfolio

Corresponding author(s): Waldherr, Mikula, Bintinger

Last updated by author(s): Feb 18, 2026

Reporting Summary

Nature Portfolio wishes to improve the reproducibility of the work that we publish. This form provides structure for consistency and transparency in reporting. For further information on Nature Portfolio policies, see our Editorial Policies and the Editorial Policy Checklist.

Statistics

For all statistical analyses, confirm that the following items are present in the figure legend, table legend, main text, or Methods section.

n/a	Confirmed	
☐	☒	The exact sample size (*n*) for each experimental group/condition, given as a discrete number and unit of measurement
☐	☒	A statement on whether measurements were taken from distinct samples or whether the same sample was measured repeatedly
☐	☒	The statistical test(s) used AND whether they are one- or two-sided<br>*Only common tests should be described solely by name; describe more complex techniques in the Methods section.*
☒	☐	A description of all covariates tested
☐	☒	A description of any assumptions or corrections, such as tests of normality and adjustment for multiple comparisons
☐	☒	A full description of the statistical parameters including central tendency (e.g. means) or other basic estimates (e.g. regression coefficient) AND variation (e.g. standard deviation) or associated estimates of uncertainty (e.g. confidence intervals)
☐	☒	For null hypothesis testing, the test statistic (e.g. F, t, r) with confidence intervals, effect sizes, degrees of freedom and P value noted<br>*Give P values as exact values whenever suitable.*
☒	☐	For Bayesian analysis, information on the choice of priors and Markov chain Monte Carlo settings
☒	☐	For hierarchical and complex designs, identification of the appropriate level for tests and full reporting of outcomes
☒	☐	Estimates of effect sizes (e.g. Cohen's d, Pearson's r), indicating how they were calculated

Our web collection on statistics for biologists contains articles on many of the points above.

Software and code

Policy information about availability of computer code

Data collection	UV-Vis absorbance and fluorescence measurements were performed using CLARIOstar (BMG Labtech), Synergy H1 (BioTek), and NanoDrop OneC (Thermo Fisher Scientific) spectrophotometers. UHPLC and HPLC-MS analyses were conducted on a Shimadzu Nexera X2 system (LC-30AD, SIL-30AC, CTO-20AC, DGU-20A5/3) with SPD-M20A PDA and LCMS-2020 MS detection. HRMS data were acquired on Agilent 6230 LC-TOF and Agilent 6545 Q-TOF instruments and analyzed using Agilent MassHunter Qualitative Analysis v10.0. Electrochemical measurements and iontronic device operation were performed using an OctoStat30 potentiostat (Ivium Technologies). Stopped-flow kinetics were measured on an SX20-LED instrument (Applied Photophysics). NMR spectra (^{1}H and ^{13}C) were recorded on a Bruker Ascend 600 MHz spectrometer and processed using Bruker TopSpin. SDS-PAGE gels were run on a Bio-Rad PowerPac HC system, imaged with a Bio-Rad ChemiDoc system, and quantified using ImageJ v1.54p. Kinetic fitting, IC50 determination, and statistical analysis were performed using GraphPad Prism v10.
Data analysis	Data analysis was performed using GraphPad Prism 10 (GraphPad Software) for curve fitting, including IC50 determination, kinetic regression analysis, and statistical testing. SDS-PAGE band intensities were quantified using ImageJ v1.54p.UHPLC and LC-MS data were processed using Shimadzu LabSolutions software (Nexera X2 platform). High-resolution mass spectrometry data were analyzed using Agilent MassHunter Workstation Qualitative Analysis v10.0.NMR spectra were processed, integrated, and assigned using Bruker TopSpin.

For manuscripts utilizing custom algorithms or software that are central to the research but not yet described in published literature, software must be made available to editors and reviewers. We strongly encourage code deposition in a community repository (e.g. GitHub). See the Nature Portfolio guidelines for submitting code & software for further information.

Data

Policy information about availability of data

All manuscripts must include a data availability statement. This statement should provide the following information, where applicable:

- Accession codes, unique identifiers, or web links for publicly available datasets
- A description of any restrictions on data availability
- For clinical datasets or third party data, please ensure that the statement adheres to our policy

All data supporting the findings of this study are available within the article and its supplementary files. Processed and raw data files generated in this study, including quantitative datasets underlying the figures and raw NMR spectroscopy data, have been deposited in the public institutional repository of TU Wien (TU Wien Research Data) under DOI 10.48436/vxpen-9kg70 (https://doi.org/10.48436/vxpen-9kg70). Any additional requests for information can be directed to, and will be fulfilled by, the corresponding authors. Source data are provided with this paper.

Research involving human participants, their data, or biological material

Policy information about studies with human participants or human data. See also policy information about sex, gender (identity/presentation), and sexual orientation and race, ethnicity and racism.

Reporting on sex and gender	Not applicable.
Reporting on race, ethnicity, or other socially relevant groupings	Not applicable.
Population characteristics	Not applicable.
Recruitment	Not applicable.
Ethics oversight	Not applicable.

Note that full information on the approval of the study protocol must also be provided in the manuscript.

Field-specific reporting

Please select the one below that is the best fit for your research. If you are not sure, read the appropriate sections before making your selection.

☒ Life sciences ☐ Behavioural & social sciences ☐ Ecological, evolutionary & environmental sciences

For a reference copy of the document with all sections, see nature.com/documents/nr-reporting-summary-flat.pdf

Life sciences study design

All studies must disclose on these points even when the disclosure is negative.

Sample size	No formal a priori power calculations were performed. Sample sizes were selected based on prior experience with iontronic delivery systems, preliminary optimization experiments, and established practice in comparable physicochemical and cell-based studies.For physicochemical and device characterization experiments, including tetrazine delivery quantification, charge-delivery correlation, step-function operation, and bead-based BSA release, n = 4-8 independent experiments per condition were performed. These replicate numbers are typical for iontronic and electrochemical device studies and were chosen to capture device-to-device variability and enable quantitative regression analysis.For cell-based viability assays, n = 6-12 independent samples per condition were used, with larger control groups where appropriate. These sample sizes are standard for 96-well plate assays and were selected to ensure reliable estimation of mean values and variance under the observed effect sizes.Across all experiments, replicate numbers were sufficient to quantify variability (mean ± SD), perform regression analyses, and apply parametric statistical testing as implemented in GraphPad Prism 10.
Data exclusions	Data were only excluded when there was a clear technical failure of the iontronic device, predefined as reaching the maximum voltage limit of 10 V, indicating non-functional tetrazine delivery. This exclusion criterion, and the affected device trace, are documented in Supplementary Fig. S13. No other data were excluded.
Replication	All key experiments were independently replicated. Click-to-release kinetics and stopped-flow measurements were performed in sextuplicate (n = 6 independent measurements per condition). Iontronic device operation experiments were replicated across independent devices (typically n = 4-8 independent devices per condition, depending on the experiment). Cell-viability assays were performed with n = 6-12 independent wells per condition and were repeated in independent experiments. Replication details for each experiment are provided in the figure legends and Supplementary Figures. Results were consistent across independent replicates, and no unexplained discrepancies between experiments were observed.
Randomization	Randomization was not relevant to this study. Treatment conditions were assigned to predefined wells or devices in cell culture plates and device holders. All wells within a given experiment were treated according to their assigned condition, and there was no allocation of individual animals or human participants.

Blinding

Blinding was not performed. Data acquisition and analysis relied on objective readouts from plate readers, HPLC, and gel densitometry, and treatment conditions were unambiguously defined by device operation parameters and compound concentrations. Given the in vitro nature of the experiments and the clear signal separation, the lack of blinding is unlikely to bias the results.

Reporting for specific materials, systems and methods

We require information from authors about some types of materials, experimental systems and methods used in many studies. Here, indicate whether each material, system or method listed is relevant to your study. If you are not sure if a list item applies to your research, read the appropriate section before selecting a response.

Materials & experimental systems

n/a	Involved in the study	
☒	☐	Antibodies
☐	☒	Eukaryotic cell lines
☒	☐	Palaeontology and archaeology
☒	☐	Animals and other organisms
☒	☐	Clinical data
☒	☐	Dual use research of concern
☒	☐	Plants

Methods

n/a	Involved in the study	
☒	☐	ChIP-seq
☒	☐	Flow cytometry
☒	☐	MRI-based neuroimaging

Eukaryotic cell lines

Policy information about cell lines and Sex and Gender in Research

Cell line source(s)

Human glioblastoma (GBM) cell line U-87, obtained from MUG cell bank (Graz, Austria, #300367, CLS).

Authentication

Cell line identity was authenticated by short tandem repeat (STR) profiling to confirm genetic identity.

Mycoplasma contamination

The U87 cell line was routinely tested for mycoplasma contamination using standard detection assays and was negative during the experiments reported in this study.

Commonly misidentified lines
(See ICLAC register)

No commonly misidentified cell lines were used. Only U87 human GBM cells from the MUG cell bank were used in this study.

Plants

Seed stocks

No live animals or other research organisms were used in this study.

Novel plant genotypes

This study did not involve human participants, human tissue, or clinical data.

Authentication

This study did not involve human participants, human tissue, or clinical data.
